# QSAR Modeling and Biological Testing of Some 15-LOX Inhibitors in a Series of Homo- and Heterocyclic Compounds

**DOI:** 10.3390/molecules29235540

**Published:** 2024-11-23

**Authors:** Veronika Khairullina, Yuliya Martynova, Matvey Kanevsky, Irina Kanevskaya, Yurii Zimin, Leonid Maksimov

**Affiliations:** 1 Institute of Chemistry and Protection in Emergency Situations, Ufa University of Science and Technology, 50076 Ufa, Russia; martynovayuz@uust.ru (Y.M.); ziminyus@uust.ru (Y.Z.); leomaxcer@gmail.com (L.M.); 2 Faculty of Biology, Saratov State University, 410012 Saratov, Russia; matvejkanev@mail.ru; 3 Institute of Chemistry, Saratov State University, 410012 Saratov, Russia; irinastrashilina@mail.ru

**Keywords:** inhibitors of 15-lipoxygenase, 15-LOX, QSAR models, GUSAR 2019 program, QNA descriptors, MNA descriptors, structure–activity relationships

## Abstract

This paper examines the quantitative structure–inhibitory activity relationship of 15-lipoxygenase (15-LOX) in sets of 100 homo- and heterocyclic compounds using GUSAR 2019 software. Statistically significant valid models were built to predict the IC50 parameter. A combination of MNA and QNA descriptors with three whole molecular descriptors (topological length, topological volume and lipophilicity) was used to develop 18 statistically significant, valid consensus QSAR models. These compounds showed varying degrees of inhibition of the catalytic activity of 15-LOX: the range of variation in the pIC_50_ value was 3.873. The satisfactory coincidence between the theoretically calculated and experimentally determined pIC_50_ values for compounds TS1, TS2 and **1–8** suggests the potential use of models M1–M18 for the virtual screening of virtual libraries and databases to find new potentially efficient inhibitors of 15-LOX.

## 1. Introduction

Lipoxygenases (EC 1.13.11.12, LOX) are oxidoreductases with iron or manganese as a cofactor, and they are the most important enzymes in biological systems. They are found in mammals, plants, fish, mosses, bacteria, yeasts, corals, algae and fungi. LOXs catalyze the oxidation of free and esterified polyunsaturated fatty acids (PUFAs) containing one or more (1Z, 4Z)-penta-1,4-diene systems into hydroperoxides, which are then metabolized into various signaling compounds such as leukotrienes and lipoxins in animals, prostaglandin-like molecules in corals, the volatile substances of green leaves, jasmonic acids in plants and lactones in microorganisms [1,2,3,4,5,6]. The LOX superfamily is classified based on their regiospecificity into 5-, 8-, 9-, 10-, 12-, 13-, 15-, fusion-, mini- and Mn-LOX. The specificity of LOXs affects the number of the carbon atom in the PUFA molecule, which is attacked by an oxygen molecule followed by the formation of hydroperoxides. Subsequently, the hydroperoxides formed are converted into ketones, aldehydes and alcohols by other enzymes [1,2,3,4,5,6,7]. Of the 10 LOX types mentioned above, the 5-, 8-, 9-, 10-, 12-, 13-, 15- LOXs are the so-called classic LOXs. Among these classical LOXs, the 9- and 13-LOXs are the most important enzymes in plants, while 5-, 12- and 15-LOX are predominantly found in animals [1,8,9]. The catalytic center of all seven families of classical LOXs (5-, 8-, 9-, 10-, 12-, 13-, and 15-LOX) contains “non-heme” iron Fe(III) in an activated state [1,2,3]. The mechanism of PUFA oxidation under the action of LOXs has been extensively studied and described in the scientific literature, e.g., in [4,5,6,7].

The relevance of the search for 15-LOX inhibitors is due to the pathophysiological effect of the products of the oxidative metabolism of PUFAs under the action of this enzyme on the organism of animals and humans. Thus, arachidonic acid metabolites formed with the participation of 15-LOX in various types of the cells and organs of animals and humans are involved in the development of many diseases, including atherosclerosis, hypertension, diabetes, obesity and neurodegenerative disorders [7,8]. In addition, 15-LOX inhibitors have been shown to be more efficient than COX inhibitors in suppressing the growth of epithelial cancer cells, regardless of the expression status of each enzyme [9,10].

To date, several types of inhibitors of the catalytic activity of LOXs are known: antioxidants and free radical acceptors (a); chelating agents (b); non-competitive redox inhibitors (c); and combinations of chelators and reducing agents (d) [11]. All of these are used in biochemistry and in the food industry to regulate PUFA metabolism under the action of LOX isoforms. However, most of these bioactive agents are 5-LOX inhibitors. Compared to 5-LOX inhibitors, the 15-LOX inhibitors are covered much less in the scientific literature. Therefore, the search for 15-LOX inhibitors is an urgent task for the medical and pharmaceutical chemistry. It should be noted that soybean 15-LOX (15-sLOX) has been used as a model for 5-hLOX due to their similarity in structure and mechanism of action [12,13,14].

Currently, QSAR/QSPR methods are actively used in the development of lead compounds and drugs based on them. These methods make it possible to solve a wide range of important tasks: (1) the unbiased search for potential bioactive compounds in virtual libraries; (2) the extension of the scope of application of active components of known drugs; (3) the evaluation of the side effects and toxicity of potential drugs and new bioactive compounds; and (4) the molecular design of new potentially low-toxicity bioactive compounds based on the structures of known drugs and hit compounds.

The application of QSAR approaches at the preclinical phase can significantly reduce the time and material costs in the targeted development of new potential drugs, as various researchers have repeatedly noted [14,15,16]. The use of QSAR/QSPR methods to solve the above problems is widely reported in the scientific literature, including research articles, scientific reviews and monographs. Every year, the well-known software packages implementing these methods are improved and new ones are developed [17].

Among the many QSAR/QSPR methods whose classification is based on the choice of certain types of descriptors and machine learning methods for constructing mathematical equations [14,15,16], the 2D- and 3D-QSAR methods are the most popular. The calculation of descriptors in these methods is based on the structural formulae and molecular diagrams of chemical compounds (2D-QSAR) and on potentially bioactive conformations of chemical structures (3D-QSAR) [14,18,19]. The choice of one of these methods depends mainly on the objectives of a particular study. These 2D-QSAR methods, together with 3D- to 6D-QSAR methods, can be used to model biological activity where there is a need to find compounds that can either increase or decrease the enzymatic specificity of certain proteins. The lack of reliable crystallographic data on potentially biologically active conformations of organic compounds in the active centers of proteins, such as in the case of 15-LOX inhibitors, increases the demand for 2D-QSAR methods over all other methods for modeling enzyme specificity. It should be understood that the application of 2D-QSAR methods is not limited to modeling biological activity. These methods can be successfully applied to model the physicochemical properties of organic compounds, as noted in our previous work and in the work of others.

GUSAR 2019 (General Unrestricted Structure–Activity Relationships) is among the programs that allow the calculation of physicochemical and structural descriptors, with the subsequent selection of the most significant ones and the construction of consensus QSAR/QSPR models based on them. Previous versions of this program are known as GUSAR 2013 and GUSAR 2011, etc. [20,21,22,23,24,25,26,27,28,29]. The demo version of the latter can be found at www.way2drug.com (accessed on 2 November 2024). Regardless of the version, this program has proved its worth in modeling different types of biological activity and a number of physicochemical properties (lipophilicity, biotransformation factor, antioxidant activity) in a number of heterogeneous organic compounds, as reported both by its developers and by us in previous works [30,31,32,33].

**Figure 1:**
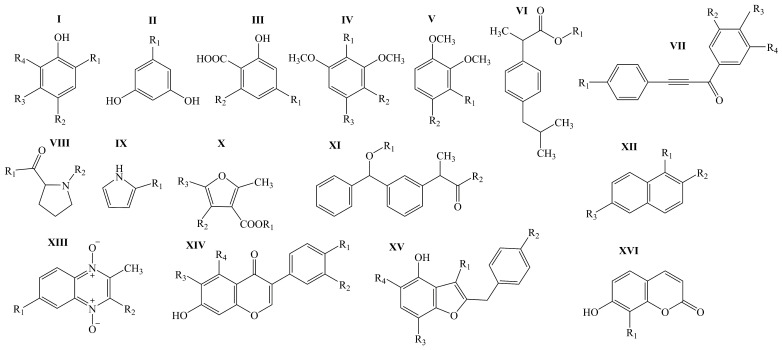
General structural formulas of modeled 15-LOX inhibitors based on a series of derivatives of phenol (I), resorcinol (II), anacardic acid (III), dimethoxybenzene (IV,V), alkyl ester of 2-(4-isobutylphenyl) propionic acid (VI), 1, 3-diarylprop-2-yn-1-one (VII), proline (VIII), pyrrole (IX), alkyl ester of 2-methylfuran-3-carbonyl acid (X), ketoprofen (XI), naphthalene (XII), 1,4-di-*N*-oxide of quinoxaline (XIII), isoflavone (XIV), 4-hydroxy-2-(phenylmethyl)benzofuran (XV) and coumarin (XVI) [34,35,36,37,38,39,40,41,42,43,44,45,46,47,48,49].

The purpose of the present work was to study the quantitative structure–activity relationship of 15-LOX inhibitors in a series of homo- and heterocyclic compounds with common structural formulae I–XVI (Figure 1) [34,35,36,37,38,39,40,41,42,43,44,45,46,47,48,49] using the GUSAR 2019 program and to build statistically significant valid prediction models for the pIC_50_ parameter designed to search for new potentially efficient 15-LOX inhibitors in virtual libraries and databases.

## 2. Results and Discussion

### 2.1. Prediction of the Numerical Values of the IC_50_ Parameter Using the GUSAR 2019 Program

As a result of QSAR modeling based on the consensus approach implemented in the GUSAR 2019 software, eighteen consensus models, M1–M18, were generated. All of these models are designed to predict the numerical values of the pIC_50_ parameter for LOX inhibitors. The difference between these models lies in the choice of different types of descriptors and the number of partial regression relationships constructed from them. The descriptive power characteristics of these consensus models are shown in Table 1. They were automatically calculated in GUSAR 2019 software based on a comparison of the experimental values of the pIC_50_ parameter for the LOX inhibitors and the values predicted by these models. In our previous works [30,31,32,33], we explained that the coefficients of determination $R^{2}$, $Q_{LMO}^{2}$ and values of standard deviation SD and F criteria presented in Table 1 are averages calculated taking into account all partial regression models included in each of the Mi consensus models.

The data presented in Table 1 allow us to use $\bar{R_{TrSi}^{2}}$ to conclude that all the QSAR consensus models, M1–M18, constructed by us feature an acceptable stability, since the A parameter for them is smaller than the value of 0.3 allowed in the scientific literature [50]. The consensus models M1–M3 and M10–M12 have the highest stability, as they provide small A values (A < 0.1). The SCR method was used to select the descriptors for their construction. The consensus models M7–M9 and M16–M18, which were constructed using the Both method for descriptor selection, show an acceptable level of stability (A ⩽ 0.2). The consensus models M5–M6 and M13–M15 have the lowest stability, with the exception of M4. The selection of descriptors in the construction of these models was performed using the RBF-SCR method. Thus, the data in Table 1 allow us to conclude that, in all cases, the nature of the descriptors on which the consensus models M1–M18 were built do not play a decisive role in their stability. At the same time, the method of descriptor selection is a significant factor that affects the stability of these models.

It should be noted that the statistical characteristics (average $R^{2}$ ($\bar{R^{2}}$), average $Q^{2}$($\bar{Q^{2}}$), average F ($\bar{F}$), average $Q_{LMO}^{2}$ ($\bar{Q_{LMO}^{2}}$), average SD ($\bar{SD}$)) whose calculation is provided by GUSAR 2019 do not allow a detailed assessment of the descriptive ability of QSAR models, while an assessment of the predictive ability of QSAR models is not performed by this program at all. Therefore, metrics based on different types of R^2^ coefficients of determination ($R^{2}$, $R_{0}^{2}$, average $R_{m}^{2}$ ($\bar{R_{m}^{2}}$), $Q_{F1}^{2}$_,_ $Q_{F2}^{2}$, average $R_{mTSi}^{2}$ ($\bar{{R_{m}^{2}}_{TSi}}$), CCC) were additionally used to objectively assess the descriptive and predictive ability of the M1–M18 QSAR models. In addition, metrics designed to evaluate the prediction errors of the pIC_50_ values (RMSE, MAE, SD) were used to determine the true prediction quality index of the pIC_50_ parameter based on the M1–M18 models for the test sets TS1–TS2 of the compounds [51,52]. The calculation of all these criteria was performed using Xternal Validation Plus 1.2 software [53]. The formulae used in this program to calculate all the above criteria are given in Table S1 in the Supplementary Materials. The same software was used to check the models for systematic errors.

**Figure 2:**
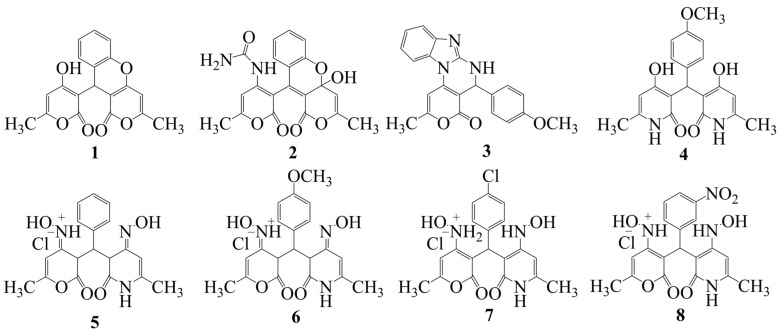
Structural formulas **1**–**8** of experimental 15-LOX inhibitors included in TS3 data set.

The process of the internal and external validation of the developed QSAR models M1–M18 was based on the structures of the training sets TrS1–TrS2 and external test set TS1, respectively. In addition, the predictive ability of the M10–M18 models was evaluated using the structures of the LOX inhibitors contained in the internal test set TS2. In addition, the predictive ability of the M3, M6, M9, M12, M15 and M18 models was evaluated by comparing the experimentally determined (see Section 2.2) and calculated values of the pIC_50_ parameter for the structures of LOX inhibitors **1–8** (see Figure 2) contained in the test set TS3.

Table 2 and Table 3 present the threshold (maximum and minimum) values of different types of coefficients of determination and prediction errors of the pIC_50_ parameter for some of the QSAR models we built. These were calculated using Xternal Validation Plus 1.2 software [53] for 100% and 95% of the structures of the 15-LOX inhibitors contained in the training sets TrS1–TrS2 and test sets TS1–TS2, respectively. Those QSAR models for which numerical data are not available in Table 2 and Table 3 occupied an intermediate position between the maximum and minimum values for all statistical features listed in this table. The data in Table 2 and Table 3 allow an objective assessment of the range of variability of different statistical criteria characterizing the descriptive and predictive ability of the M1–M18 QSAR models. Tables S2–S6 in the Supplementary Materials present the full set of criteria calculated using Xternal Validation Plus 1.2 software for the TrS1–TrS2 and TS1–TS2 sets, considering both 100% and 95% of the 15-LOX inhibitor structures contained therein.

The data from Table 2 allow us to conclude that all the models M1–M18 showed high predictive power.

**Figure 3:**
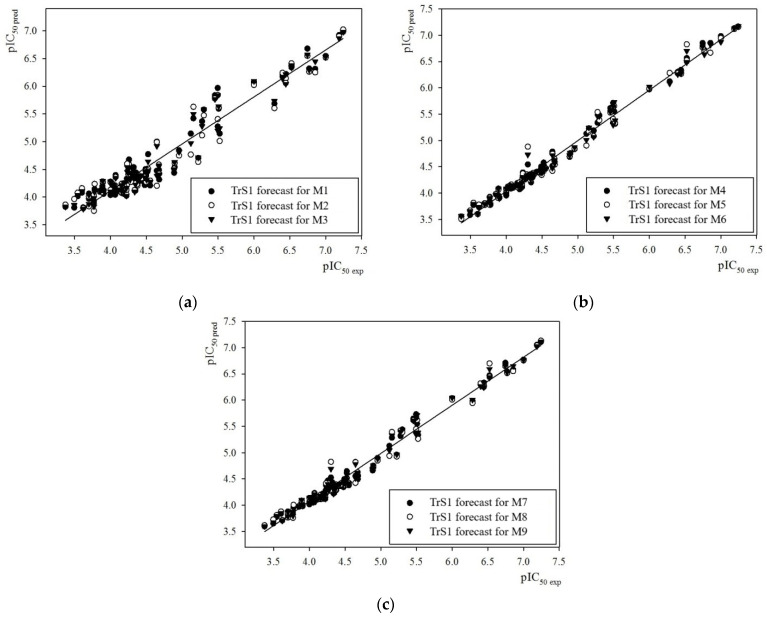
Comparison of experimental pIC_50_ values (pIC_50_^exp^) with those predicted (pIC_50_^pred^) by models M1 to M9 for 15-LOX inhibitors contained in training set TrS1 using three methods: SCR (**a**), RBF-SCR (**b**) and Both (**c**).

Figure 3 shows correlations between the experimental and calculated values of the pIC_50_ parameter for the 15-LOX inhibitors contained in the TrS1 set, as an example of the high predictive power of our QSAR models M1–M9.

In this case, according to Table 2, the best descriptive ability was provided by the M4, M13 and M15 QSAR models built using RBF-SCR as the descriptor selection method. The lowest accuracy in reproducing the experimental data contained in the TrS1–TrS2 sets was demonstrated by the M2 and M10 models, in which the descriptors were selected using the SCR method. In particular, the M2 model had the smallest numerical values of different types of coefficients of determination and the largest values of prediction errors of the pIC_50_ parameter for the structures contained in the TrS1 set. At the same time, the M4 model showed the largest numerical values of different types of coefficients of determination and the smallest error values. This conclusion holds true for both 100% and 95% of the data contained in the TrS1 set.

The conclusions drawn from the analysis of the goodness of fit of the M10–M18 models built on the TrS2 set are less clear. For example, a detailed analysis of the different types of coefficients of determination and prediction errors shows small differences in the estimation of the descriptive ability of the M10–M18 models depending on the completeness of inclusion of the experimental data (100% and 95% of the data) contained in the TrS2 set. In fact, if 100% of the data in TrS2 are considered, the maximum coefficients of determination $R_{0}^{2}$, $R_{0}^{2\prime }$, $\bar{R_{m}^{2}}$ and CCC and the minimum ${\Delta R}_{m}^{2}$ coefficient are provided by the M13 model. The same model, based on the data in the tables, is characterized by minimum values of error and standard deviation in the prediction of the pIC_50_ parameter for the structures of the 15-LOX inhibitors included in the TrS2 set. The maximum value of the $R^{2}$ coefficient is given by the M15 model.

If 95% of the data of the training set TrS2 is taken into account, the M13 model showed the best descriptive power even on the basis of a smaller set of criteria. In fact, it was characterized by the largest values of coefficients of determination such as $R^{2}$, $R_{0}^{2}$ and CCC, along with the minimum value of the ${\Delta R}_{m}^{2}$ coefficient. The M15 model gave the largest values of the coefficients $R_{0}^{2\prime }$ and mean $R_{m}^{2}$, in combination with the smallest values of the RMSE, B parameter and standard deviation (SD). At the same time, the minimum value of MAE was observed for the M14 model based on 95% of the data of the TrS2 training set.

The data of Table 3 and Tables S4–S6 in the Supplementary Materials allow us to conclude that almost all the QSAR models built by us are characterized by a moderate predictive ability and the absence of systematic errors in predicting the target property for the structures of 15-LOX inhibitors contained in test sets TS1–TS2. The highest numerical values of different types of coefficients of determination in predicting the pIC_50_ parameter for 100% of the 15-LOX inhibitor structures contained in TS1 were demonstrated by the M14 model. The same model had the smallest value of RMSE. The smallest values of MAE, SD value and B parameter for 100% of the data in TS1 were provided by the M13 and M17 models, respectively.

The worst results for all the statistical criteria described above were observed for the M9 and M12 models. For example, the M9 model was characterized by the minimum values of then $R_{0}^{2}$, $R_{0}^{2\prime }$, $\bar{R_{m}^{2}}$ and CCC criteria. The M12 model gave the minimum values of the n $R_{0}^{2}$, $R_{0}^{2\prime }$, $\bar{R_{m}^{2}}$ and CCC criteria, while the M12 model provided the minimum values of the $R^{2}$, $R_{0}^{2}$, $Q_{F1}^{2}$ and $Q_{F2}^{2}$ criteria, combined with the maximum value of RMSEP. However, the M18 model was characterized by the maximum value of the ${\Delta R}_{m}^{2}$ coefficient. The highest value of MAE in predicting the pIC_50_ parameter for 100% of the TS1 structures was demonstrated by the M3 model. The M5 model gave the maximum values of the SD and B parameters.

The results of evaluating the predictive ability of QSAR models M10–M18 using 100% of the data of test set TS2 were more unambiguous. The maximum values of the different types of determination coefficients, along with the minimum values of the prediction errors of the pIC_50_ parameter, in this case belonged to the M13 model. The minimum values of the determination coefficients in combination with the maximum values of the prediction errors of the pIC_50_ parameter for the 15-LOX inhibitors corresponded to the M11 model.

The removal of 5% of the data with the worst prediction results for pIC_50_ from TS1 and TS2 contributed to a slight increase in the numerical values of different types of coefficients of determination and a decrease in the numerical values of the prediction errors for this parameter. However, the changes in all the statistical criteria were not systematic when the data from both sets were manipulated in this way. In particular, after removing 5% of data from TS1, the highest numerical values of the coefficients of determination the $R^{2}$, $R_{0}^{2}$, $Q_{F1}^{2}$ and $Q_{F2}^{2}$, the $\bar{R_{m}^{2}}$ were obtained from the M10 model. The maximum value of the R^2′^_0_ criterion in a comparison of the experimental pIC_50_ values with the predicted ones was observed for the M1 model. The minimum values of RMSE and MAE based on 95% of the data of the TS1 set were shown by the M13 and M16 models, respectively. The lowest values of SD and B were shown by the M4 model.

Removing 5% of the data from the TS2 set had almost no effect on the generalizing conclusions. In this case, like in the case of 100% data in TS2, the M13 model provided the largest numerical values of different types of coefficients of determination and the smallest values of pIC_50_ prediction errors for the 15-LOX inhibitors, with the exception of SD, whose numerical value was the smallest for the M17 model. The minimum numerical values of different types of coefficients of determination, combined with the maximum values of pIC_50_ prediction errors, for both 100% and 95% data in TS2 were observed for the M11 model.

Based on an analysis of the numerical values of different validation criteria presented in Table 1, Table 2 and Table 3 of this section and in Tables S2–S6 in the Supplementary Materials, we can conclude that almost all the models showed a high descriptive and moderate predictive power, since they met the internal and external validation criteria described in Section 2.3. It should be noted that this condition was met for both 100% and 95% of the data contained in the TrS1–TrS2 and TS1–TS2 sets.

### 2.2. Experimental Determination of the IC_50_ Parameter Against 15-LOX for Compounds ***1**–**8***

**Figure 4:**
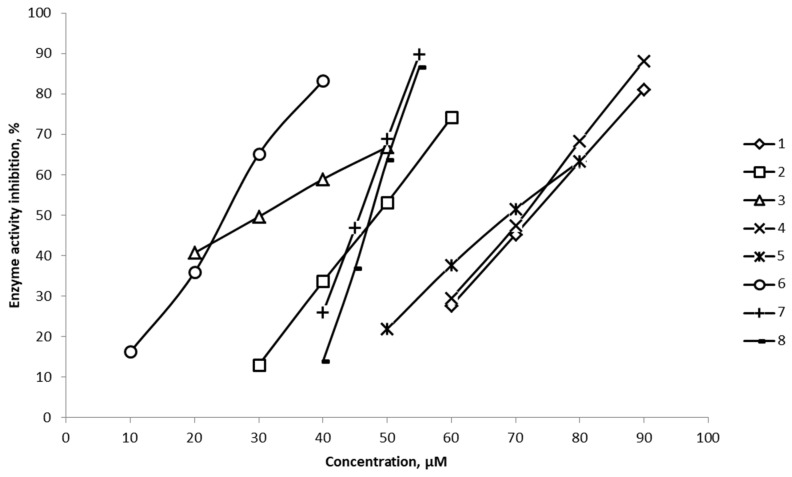
Decrease in 15-LOX activity as a function of the concentration of inhibitors **1–8**.

The results of the in vitro analysis of the inhibitory activity of a series of 2H-(benzo)pyran-2-one derivatives, **1–8**, against 15-LOX are presented in Table 4 and Figure 4.

IC_50_ values were determined by linear interpolation between the points closest to 50% inhibition (Figure 4).

The inhibitory activity against 15-LOX was experimentally determined for compounds **1**–**8**. The values of semi-efficient inhibitory concentration are in the range of 24–73 μmol/L, which allows us to classify these compounds as moderate inhibitors of the enzyme.

As can be seen from the plot (Figure 4), a sharp change in inhibitory activity was observed in a narrow concentration range for compounds **7**, **8**, which can serve as a basis for the assumption that the enzyme is highly sensitive to these inhibitors.

### 2.3. Evaluation of the Predictive Ability of the M3, M6, M9, M12, M15 and M18 Models Based on Compounds ***1**–**8*** in the Test Set TS3

Subsequently, the consensus models M3, M6, M9, M12, M15 and M18 were used to predict the numerical values of the pIC_50_ parameter for compounds **1–8** from the TS3 set. The results of these calculations in comparison with the experimental values of the pIC_50_^exp^ parameter and the 2·RMSEP criterion, which corresponded to 100% and 95% of the data from the TS1 and TS2 sets, respectively, for these compounds are shown in Table 5 and Table 6. In selecting the numerical values of the 2·RMSEP criterion corresponding to the M3, M6, M9, M12, M15 and M18 models for subsequent comparison with the ΔpIC_50_ values, which are in fact equal to the modulus of the difference between the experimental and predicted values of the pIC_50_ parameter, we focused on the minimum value of this parameter (see Table 5). It should be noted that all the tested compounds were within the range of applicability of the M3, M6, M9, M12, M15 and M18 models. These models were selected for the prediction of the pIC_50_ parameter for 15-LOX inhibitors **1**–**8** because each of these consensus models included 320 partial regression relationships, which makes it possible to take into account the structural characteristics of each of the tested compounds in the most objective and complete way. In addition, it was of scientific interest to explore the applicability of these models with a satisfactory and worst case predictive performance, judging by the statistical criteria derived from TS1–TS2, to predict the target property for new compounds not included in the modeling.

The data in Table 5 and Table 6 allow us to conclude that almost all numerical values of pIC_50_ for compounds **1**–**8** predicted by the M3, M6, M9, M12, M15 and M18 models fall within the 95% confidence interval equal to ±2·RMSE, i.e., the difference between the predicted and experimentally determined values of the pIC_50_ parameter for 15-LOX inhibitors **1–8** does not numerically exceed the minimum value of the 2·RMSEP criterion for each of the models (see Table 5 and Table 6). The M3 model showed the highest prediction error by this criterion when the pIC_50_ parameter was predicted for compound **4**, but even in this case the difference between the experimental and theoretically predicted values of the pIC_50_ parameter fell within the 2·RMSEP range. However, the same model (M3) showed the smallest prediction error of the pIC_50_ parameter for compound **8**. This demonstrates the good predictive ability and correctness of our constructed models, as well as the applicability of the GUSAR 2019 program for modeling 15-LOX inhibitors.

Thus, all the QSAR consensus models M1–M18 are characterized by a high descriptive and moderate predictive power when comparing experimental and predicted values of pIC_50_ based on TrS1 and TrS2 training set structures, external and internal test sets TS1 and TS2, and compounds **1**–**8**. These models can be used for the virtual screening of virtual libraries and databases to search for new 15-LOX inhibitors in the series of homo- and heterocyclic compounds with common structural formulae I-XVI.

## 3. Research Methods

The simulation procedure was performed for the compounds whose formulas are shown in Figure 1.

### 3.1. The Methodology of the Computational Experiment

QSAR modeling of 15-LOX inhibitors with general structural formulae I–XVI (Figure 1) was performed using the GUSAR 2019 (General Unrestricted Structure–Activity Relationships) computer program [23,27].

**Figure 5:**
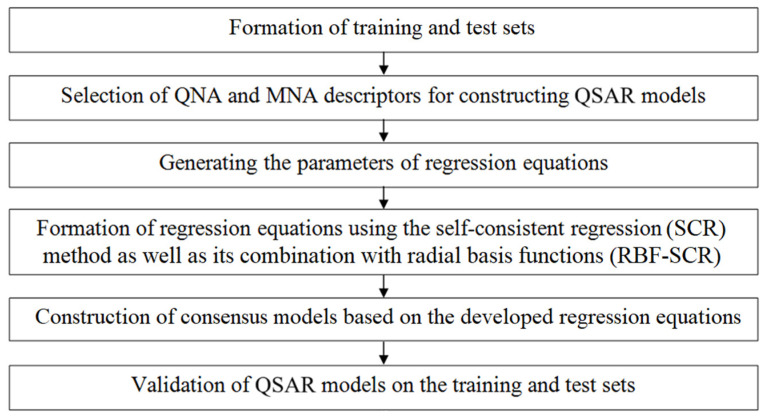
Schematic representation of GUSAR algorithm.

The QSAR models were built in several steps that are the basis of the operation of this program and have been described in detail in our previous work [30,31,32,33]. The stages of QSAR model building are shown schematically in Figure 5.

### 3.2. Formation of the Training and Test Sets

**Figure 6:**
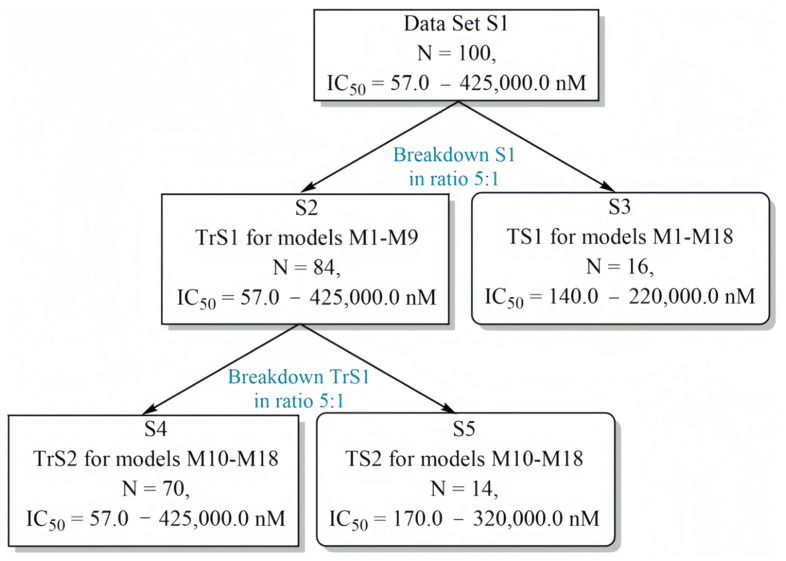
Chart of constructing the training and test sets and the design of the QSAR consensus models M1–M18 (S denotes “set”; TrS and TS are “training and test sets”, respectively; N is the number of compounds included in the corresponding sets and arrays). Designations: (1) S1 is all data sets; (2) S2 is the training set TrS1 for models M1–M9; (3) S3 is the external test set TS1 for models M1–M18; (4) S4 is the training set TrS2 for models M10–M18; (5) S5 is the internal test set TS2 for models M10–M18.

The training set TrS1 and test set TS1 were generated from the structure set S1. The structure set S1 contained 100 15-LOX inhibitors with their corresponding pIC_50_ values. The training set TrS2 and test set TS2 were formed based on the structures contained in training set TrS1. Figure 6 shows a scheme that clearly illustrates the strategy of forming the training and test sets TrS1–TrS2 and TS1–TS2.

The pIC_50_ parameter for each compound included in the data set S1 (and the training TrS1–TrS2 and test sets TS1–TS2 generated from it) was calculated as the negative decimal logarithm of its corresponding IC_50_ value (in mol/L). Numerical IC_50_ values for the 15-LOX inhibitors being modeled were measured experimentally and are given in [34,35,36,37,38,39,40,41,42,43,44,45,46,47,48,49]. The complete list of organic compounds from which the data set S1 was generated, with their corresponding pIC_50_ characteristics, is presented in Table S8 in the Supplementary Materials.

QSAR models M1–M9 were built using the TrS1 training set, which contained 84 structures of 15-LOX inhibitors with their corresponding values of the pIC_50_ parameter. To test the predictive ability of the M1–M9 models, a test set TS1 containing 16 15-LOX inhibitors with their corresponding values of the pIC_50_ parameter was used. Both sets were obtained by partitioning the data set S1, in which all compounds were previously ranked in ascending order of the pIC_50_ parameter, in a 5:1 ratio. The structures were partitioned into the training set TrS1 and the test set TS1 by transferring every sixth compound from the data set S1 to TS1. The remaining 84 structures of the 15-LOX inhibitors were used to form the training set TrS1.

The TrS2 set contained 70 15-LOX inhibitors with their corresponding values of the pIC_50_ parameter. It was intended for constructing the QSAR models M10–M18. The validity of the QSAR models M10–M18 was tested using the TS2 set. Both sets, TrS2 and TS2, were generated on the basis of TrS1. The same principle was used to generate the training set TrS1 and the test set TS1 from the data set S1. The characteristics of the training sets TrS1 and TrS2 and the test sets TS1 and TS2 are shown in Table 7 and Table 8, respectively. The data in these tables indicate that the compounds of the training and test sets are fairly evenly distributed over the entire range of pIC_50_ variation. At the same time, the range of variation of the pIC_50_ parameter for the 15-LOX inhibitors included in training sets TrS1–TrS2 and test sets TS1–TS2 exceeds the ΔpIC_50_ value of 3 (i.e., ΔpIC_50_ > 3), which determines the correctness of the further QSAR modeling process [50]. In addition, as can be seen from Figure 1, the training sets are characterized by rather a high degree of molecular diversity.

The structures of the compounds in the training and test sets TrS1–TrS2 and TS1–TS2 were plotted in Marvin Sketch 23.4 software [54] and then converted to SDF format using Discovery Studio Visualizer v24.1 software [55].

### 3.3. Building QSAR Models

The M1–M18 QSAR models were built on the basis of two types of substructural descriptors of atomic neighborhoods, QNA (Quantitative Neighborhoods of Atoms) and MNA (Multilevel Neighborhoods of Atoms), and three types of whole molecule descriptors (topological length, topological volume and lipophilicity). These types of descriptors are automatically calculated by the GUSAR 2019 program. At the same time, the QNA and MNA descriptors are unique characteristics of molecules, and their calculation is available in different versions of the GUSAR software (GUSAR 2011, GUSAR 2013 and GUSAR 2019). The ideology of calculating QNA and MNA descriptors was proposed by Professor V.V. Poroikov’s research team. It is described in detail in the Supplementary Materials and in a number of articles [20,21,22,23,24,25,26,27,28,29]. The rather complicated mathematical apparatus used for calculating QNA descriptors complicates their physical interpretation. Therefore, they are not explicitly displayed in the section dealing with calculations.

The MNA descriptors were computed using the PASS (Prediction of Activity Spectra for Substances) algorithm, which predicts approximately 6400 “biological activities” with an accuracy threshold of an average prediction of at least 95%. These descriptors are generated based on the structural formulae of the chemical compounds without using any pre-compiled list of structural fragments [20,21,22,23,24,25]. They are generated as a recursively defined sequence:

- The zero-level MNA descriptor for each atom is mark A of the atom itself;
- Any next-level MNA descriptor for the atom is substructure notation A (D_1_D_2_ … D_i_ …), where D_i_ is the previous-level MNA descriptor for the i–th immediate neighbor of the atom A.

The neighbor descriptors D_1_ D_2_ … D_i_ … are arranged in a unique manner. This may be, for example, a lexicographic sequence. The MNA descriptors are generated using an iterative procedure, which results in the formation of structural descriptors that include the first, second, etc., neighborhoods of each atom. The label contains not only information about the type of atom but also additional information about whether it belongs to a cyclic or acyclic system, etc.

Three methods were used to reduce the descriptor space and select the most significant descriptors:

1. Self-consistent regression (SCR) method;
2. The method of combining self-consistent regression with radial basis functions (RBF-SCR);
3. The Bath method, which combines the simultaneous use of the SCR and RBF-SCR methods in a unique way.

All three of these unique methods were also developed by Professor Poroikov’s research team and implemented in the GUSAR 2019 program for selecting the most appropriate options. A more detailed description of each method can be found in the Supplementary Materials and in the relevant papers [21,22,23,24,25].

The stability of the models was tested by using a sliding control procedure, with a 20-fold randomized release of 20% of the compounds from the training samples TS1 and TS2. Both of these procedures are automatically implemented in the GUSAR 2019 program [23,27].

Each of the eighteen final QSAR M1–M18 models was based on a consensus approach. This approach involves combining several regression equations into one model, which is carried out automatically based on the similarities between the equations.

Each of the final QSAR models, M1–M2 and M4–M5, M7–M8, M10–M11, M13–M14 and M16–M17, included 20 partial regression dependences. At the same time, the M1, M4, M7, M10, M13 and M16 models were based on QNA descriptors and three additional descriptors that describe the topological length, topological volume and lipophilicity of the modeled 15-LOX inhibitors. The M2, M5, M8, M11, M14 and M17 models were built on a similar principle, but they were based on NA descriptors, with the automatic addition of the three whole molecule descriptors described above. The M3, M6, M9, M12, M15 and M18 models each included 320 partial regression dependences. At the same time, each of these 320 particular models was built independently of each other based on the three descriptors of the entire molecule described above, with the addition of the QNA or MNA descriptors.

Due to the specifics of the calculation process, which is described in detail in the Supplemental Material, the QNA and MNA descriptors are not amenable to unambiguous physical interpretation. Therefore, the regression equations based on these descriptors are not explicitly displayed in the GUSAR 2019 program.

### 3.4. Evaluation of Descriptive and Predictive Ability of QSAR Models

The descriptive ability of the M1–M18 models was evaluated using several metrics. These included metrics based on the coefficients of determinations R2, R20, average R2m and CCC, as well as metrics estimating errors in predicting pIC_50_ values (RMS error (RMSE), mean absolute error (MAE) and standard deviation (SD)). The parameters of the predictive ability of the M1–M18 models also included metrics based on the coefficients of determination $R^{2}$, $R_{0}^{2}$, $\bar{R_{m}^{2}}$, CCC, $Q_{F1}^{2}$ and $Q_{F2}^{2}$, as well as metrics estimating errors in predicting pIC_50_ values (RMS error (RMSE), mean absolute error (MAE), standard deviation (SD)).

These statistical parameters were calculated using Xternal Validation Plus 1.2 software for 100% and 95% of the data (to account for errors) contained in the training and test samples [53]. The Supplementary Materials contain formulas for automatically calculating these criteria in this program. The internal verification of the M1–M6 models was performed using LMO cross-validation ($Q_{LMO}^{2}$), with 20% of the compounds excluded from the training sets.

The threshold values of the validation criteria for the above parameters, based on which the descriptive and predictive ability of the QSAR M1–M18 models was evaluated, are presented in Table 9.

Acceptable values of different types of determination coefficients, based on $R^{2}$, as well as ranges of variation in the MAE and RMSD values and criterion B for assessing the descriptive and predictive ability of QSAR models M1–M18, were calculated taking the recommendations from the leading scientists in the field of QSAR modeling [51,52] into account.

Thus, the permissible range of variation for the MAE parameter was estimated considering the range of variability in the pIC_50_ parameter for the compounds in training sets TrS1 and TrS2, using the following formulas:

MAE < 0.1 pIC_50_—if the criterion is met, then the models are characterized by a high predictive ability.

MAE = [0.1; 0.15} pIC_50_ —if the criterion is met, then the models are characterized by a moderate predictive ability.

MAE > 0.15 pIC_50_—if the criterion is met, then the models are characterized by a low predictive ability.

The permissible range of variation of criterion B, where B=MAE+3SD, was estimated using the following formulas:

B < 0.2 pIC_50_—if the criterion is met, then the models are characterized by a high predictive ability.

B = [0.2; 0.25] pIC_50_—if the criterion is met, then the models are characterized by a moderate predictive ability.

B > 0.25 pIC_50_—if the criterion is met, then the models are characterized by a low predictive ability.

Additionally, the predictive ability of the QSAR M1–M18 models was evaluated by comparing the predicted pIC_50_ values with the experimental values of the same parameter for the new promising 15-LOX inhibitors **1**–**8** contained in the TS3 test set (Figure 2). These compounds were missing in the S1 data set and, accordingly, did not participate in the consensus models.

### 3.5. The Technique of the Biochemical Experiment to Measure Inhibitory Activity

Substances **1**–**8** were kindly provided by the staff of the Department of Organic and Bioorganic Chemistry of Saratov State University. Compounds **1**–**8** were synthesized under the supervision of I.V. Strashilina, PhD. The methodology for the synthesis of samples **1**–**4** is described in detail in works [56,57,58]. Samples **5**–**8** were obtained using the method described in [58], with modification by replacing ammonium acetate with hydroxylamine hydrochloride [56] with a purity level of 97–98%.

Biological tests of the inhibitors of 15-LOX **1**–**8** (Figure 2) were performed at the Department of Biochemistry and Biophysics under the supervision of M.V. Kanevsky, PhD., at the Saratov State University. The inhibition of the catalytic activity of 15-lipoxygenase by the substances studied was evaluated according to the procedure reported elsewhere [59,60,61]. Quercetin, a well-known inhibitor of 15-LOX, was employed as the positive control.

For the biological tests, Lipoxidase/15-lipoxygenase (Sigma Aldrich, Darmstadt, Germany, lyophilizate, 15 million Units, CAS Number: 9029-60-1) was used. The study was carried out in borate buffer (0.2 M, pH 9.0). Linoleic acid (Sigma Aldrich, Germany, purity ≥ 95.0%, CAS Number: 60-33-3) was used as the oxidation substrate. The activity of 15-LOX in the presence of the compounds studied was evaluated by a spectrophotometric recording of changes in the concentration of 13-hydroperoxylinoleic acid, the product of oxidative transformation of linoleic acid [61], using a LEKI SS2110UV two-beam scanning spectrophotometer (CJSC LOIP, Saint-Petersburg, Russia).

The initial concentration of lipoxygenase in the sample was 167 U/mL. Its final concentration in the sample was 134 µM. In order to study the inhibitory activity of the compounds, they were added to the sample as solutions in DMSO.

To conduct the experiment, 1400 µL of a substrate solution was placed in a cell, along with 24 µL of a sample in DMSO (for the prototype) or DMSO (for the control sample). Immediately after adding 76 µL of the enzyme solution, the timer was turned on. After 90 s, the optical density (λ = 234 nm) was recorded.

The activity value obtained from the control experiment was used as a baseline, or 100%, of enzyme activity. It was measured with only the enzyme solution and solvent (DMSO) present in the cell, without any active substance.

The percentage of activity inhibition by compounds **1**–**8** was determined as a relative decrease in the optical density of the solution, using Formula (1):

(1) $$I=\frac{D_{c}{-D}_{t}}{D_{c}}\times 100\%,$$

where $D_{c}$ is the optical density index of the control sample 90 s after the start of the reaction and $D_{t}$ is the optical density index of the sample containing the test compound 90 s after the start of the reaction.

The range of working concentrations of compounds **1**–**8** was 1–100 µM.

The IC_50_ values of compounds **1**–**8** were determined using linear interpolation between the points closest to 50% inhibition by means of Microsoft Excel 2016. Enzyme activity experiments were performed in triplicate. The values are expressed as means ± SD. Student’s *t*-test was employed for the determination of statistical significance, using a p value of 0.05 or less as a criterion for significant inhibition.

## 4. Conclusions

Using the QSAR methodology implemented in the GUSAR 2019 program, a quantitative structure–inhibitory activity relationship has been found for a series of 100 15-LOX inhibitors based on a series of derivatives of phenol, resorcinol, anacardic acid, dimethoxybenzene, alkyl ester of 2-(4-isobutylphenyl)propionic acid, 1,3-diarylprop-2-yn-1-one, proline, pyrrole, alkyl ester of 2-methylfuran-3-carbonyl acid, ketoprofen, naphthalene, 1,4-di-N-oxide of quinoxaline, isoflavone, 4-hydroxy-2-(phenylmethyl)benzofuran and coumarin with the general structural formulas I–XIV. These compounds showed various degrees of inhibition of the catalytic activity of 15-LOX. The variation range of the pIC_50_ parameter was 3.873 (pIC_50_ = 3.873). Based on a combination of MNA and QNA descriptors with three whole molecule descriptors, including topological length, topological volume and lipophilicity, eighteen statistically significant and valid consensus models (M1–M18) were generated.

All the models reproduced the experimental data contained in the training samples with a high degree of accuracy. Cross-validation with a 20-fold deletion of 20% of the data from the training samples also showed good results. The reliability of the prediction of the pIC_50_ parameter based on the evaluation of this parameter for compounds of two test samples and ten subsequently experimentally studied compounds showed the moderate predictive ability of the QSAR M1–M18 models.

The satisfactory match of the theoretically calculated pIC_50_^pred^ values with the experimental pIC_50_^exp^ values for the compounds of test sets TS1–TS2 and compounds **1**–**8** opens up prospects for the application of the M1–M18 models in the virtual screening of virtual libraries and databases in the search for new potentially efficient 15-LOX inhibitors in these sources.

## Figures and Tables

**Table 1:** Statistical parameters and accuracy of the predicted pIC_50_ values of the compounds included in the training sets TrS1–TrS2 within the consensus models M1–M18. pIC_50 TrS1_ = pIC_50 TrS2_ = 3.873, pIC_50 TS1_ = 3.196, pIC_50 TS2_ = 3.275.

Training Set	Method	Model	*N* ^1^	*N_PM_*	$\bar{R_{TrSi}^{2}}$	$\bar{Q_{LMO}^{2}}$	$\bar{F}$	$\bar{SD}$	*V*	*A* ^2^
QSAR models based on QNA descriptors										
TrS1	SCR	M1	84	20	0.825	0.758	10.429	0.485	17	0.067
TrS2		M10	70	20	0.804	0.714	7.608	0.531	15	0.090
TrS1	RBF-SCR	M4	84	20	0.997	0.802	14.606	0.437	17	0.195
TrS2		M13	70	20	0.996	0.753	10.204	0.492	15	0.243
TrS1	Both	M7	84	20	0.962	0.800	13.026	0.443	17	0.162
TrS2		M16	70	20	0.959	0.759	9.264	0.491	15	0.200
QSAR models based on MNA descriptors										
TrS1	SCR	M2	84	20	0.798	0.725	8.749	0.517	16	0.073
TrS2		M11	70	20	0.825	0.741	6.444	0.512	17	0.084
TrS1	RBF-SCR	M5	84	20	0.985	0.745	11.115	0.495	16	0.240
TrS2		M14	70	20	0.982	0.725	7.267	0.518	17	0.257
TrS1	Both	M8	84	20	0.955	0.760	10.365	0.486	16	0.195
TrS2		M17	70	20	0.959	0.759	7.170	0.495	17	0.200
QSAR models based on both QNA and MNA descriptors										
TrS1	SCR	M3	84	320	0.842	0.777	8.747	0.480	17	0.065
TrS2		M12	70	320	0.842	0.766	7.067	0.499	16	0.076
TrS1	RBF-SCR	M6	84	320	0.991	0.783	11.373	0.460	17	0.208
TrS2		M15	70	320	0.99	0.769	9.189	0.480	16	0.221
TrS1	Both	M9	84	320	0.965	0.798	10.443	0.454	17	0.167
TrS2		M18	70	320	0.966	0.787	8.401	0.474	16	0.179

^1^ N is the number of structures in the training set; $N_{PM}$ is the number of regression equations used for the consensus model; $\bar{R_{TrSi}^{2}}$ is the coefficient of determination calculated for the compounds of TrSi; $\bar{Q_{LMO}^{2}}$ is the correlation coefficient calculated for the training set by cross-validation with the exception of one; $\bar{F}$ is Fisher’s criterion; $\bar{SD}$ is the standard deviation; V is the number of variables in the final regression equation. ^2^ $A=\bar{R_{TrSi}^{2}}-\bar{Q_{LMO}^{2}}$.

**Table 2:** Range of variability of statistical criteria in assessing the descriptive power of models M1–M18.

Criteria	Code of Training Set							
	TrS1				TrS2			
	100% Data of TrS1		95% Data of TrS1		100% Data of TrS2		95% Data of TrS2	
	Max	Min	Max	Min	Max	Min	Max	Min
$R^{2}$	M4	M2	M4	M2	M13, M15	M10	M15	M10
	0.990	0.932	0.993	0.942	0.986	0.923	0.991	0.934
$R_{0}^{2}$	M4	M2	M4	M2	M13	M10	M15	M10
	0.989	0.920	0.992	0.933	0.985	0.914	0.991	0.934
$R_{0}^{2\prime }$	M4	M2	M4	M2	M13	M10	M13	M10
	0.989	0.891	0.963	0.786	0.984	0.886	0.9545	0.781
$\bar{R_{m}^{2}}$	M4	M2	M4	M2	M13	M10	M13	M10
	0.964	0.811	0.971	0.837	0.959	0.813	0.969	0.832
${\Delta R}_{m}^{2}$	M2	M4	M2	M4	M10	M13	M10	M15
	0.067	0.009	0.057	0.006	0.071	0.012	0.062	0.008
CCC	M4	M2	M4	M2	M13, M15	M10	M13, M15	M10
	0.993	0.942	0.996	0.962	0.992	0.951	0.995	0.959
RMSE	M2	M4	M2	M4	M10	M13	M10	M14, M15
	0.278	0.101	0.244	0.088	0.290	0.120	0.260	0.101
MAE	M2	M4	M2	M4	M10	M13	M10	M14
	0.225	0.079	0.201	0.070	0.240	0.092	0.218	0.079
SD	M2	M4	M2	M4	M11	M13	M11	M15
	0.165	0.063	0.140	0.053	0.168	0.078	0.147	0.060
MAE+3·SD	M2	M4	M2	M4	M10	M13	M10	M15
	0.719	0.268	0.620	0.230	0.733	0.326	0.644	0.261

**Table 3:** Range of variability of statistical criteria in assessing the predictive ability of models M1–M18 using test sets TS1–TS2.

Criteria	Code of Test Set							
	TS1				TS2			
	100% Data of TS1		95% Data of TS1		100% Data of TS2		95% Data of TS2	
	Max	Min	Max	Min	Max	Min	Max	Min
$R^{2}$	M14	M9, M12	M10	M3	M13	M11	M13	M11
	0.832	0.776	0.870	0.798	0.849	0.723	0.880	0.730
$R_{0}^{2}$	M14	M12	M10	M3	M13	M11	M13	M11
	0.832	0.775	0.870	0.790	0.848	0.721	0.868	0.722
$R_{0}^{2\prime }$	M14	M9	M1	M12	M13	M11	M16	M11
	0.806	0.711	0.820	0.627	0.809	0.580	0.700	0.583
$\bar{R_{m}^{2}}$	M14	M12	M10	M3, M12	M13	M11	M13	M11
	0.872	0.832	0.903	0.866	0.894	0.805	0.914	0.843
${\Delta R}_{m}^{2}$	M14	M12	M10	M3	M13	M11	M13	M11
	0.828	0.774	0.869	0.783	0.845	0.716	0.868	0.673
CCC	M14	M9	M10	M3	M13	M11	M13	M11
	0.761	0.700	0.815	0.732	0.748	0.574	0.742	0.633
RMSEP	M1	M18	M12	M1	M11	M13	M11	M13
	0.147	0.040	0.123	0.016	0.204	0.118	0.186	0.104
MAE	M14	M9	M10	M3	M13	M11	M13	M11
	0.909	0.873	0.928	0.892	0.915	0.831	0.921	0.836
SD	M12	M14	M12	M13	M11	M13	M11	M13
	0.441	0.384	0.406	0.338	0.497	0.367	0.433	0.342
MAE+3·SD	M3	M13	M12	M16	M11	M13	M11	M13
	0.377	0.326	0.347	0.287	0.411	0.314	0.365	0.291

**Table 4:** Experimental characterization of the inhibition of 15-LOX activity in the presence of compounds **1**–**8**.

Compound	Concentration, μM	Enzyme Activity Inhibition, %	IC_50_, µmol/L
**1**	60	27.62	72.5
	70	45.25	
	80	63.30	
	90	81.12	
**2**	30	12.99	48.2
	40	33.61	
	50	53.12	
	60	74.14	
**3**	20	40.679	30.4
	30	49.593	
	40	58.907	
	50	66.821	
**4**	60	29.47	70.8
	70	47.37	
	80	68.27	
	90	88.18	
**5**	50	21.78	69.6
	60	37.64	
	70	51.49	
	80	63.36	
**6**	10	16.21	24.9
	20	35.89	
	30	65.08	
	40	83.27	
**7**	40	25.91	45.7
	45	46.84	
	50	68.77	
	55	89.70	
**8**	40	13.88	47.4
	45	36.79	
	50	63.70	
	55	86.60	

**Table 5:** Results of parameter pIC_50_ prediction for 15-LOX inhibitors **1**–**8** by QSAR models M3, M6, M9, M12, M15 and M18.

Compound	pIC_50_^exp 1^	SCR			RBF-SCR			Both		
		Model	pIC_50_^pred^	ΔpIC_50_^2^	Model	pIC_50_^pred^	ΔpIC_50_	Model	pIC_50_^pred^	ΔpIC_50_
**1**	4.140	M3	4.323	0.183	M6	4.313	0.173	M9	4.267	0.127
		M12	4.301	0.161	M15	4.301	0.161	M18	4.249	0.109
**2**	4.317	M3	4.033	0.284	M6	4.063	0.254	M9	3.934	0.383
		M12	4.145	0.172	M15	4.166	0.151	M18	4.054	0.263
**3**	4.517	M3	4.086	0.431	M6	4.119	0.398	M9	4.081	0.436
		M12	4.054	0.463	M15	4.112	0.405	M18	4.052	0.465
**4**	4.150	M3	4.874	0.724	M6	4.836	0.686	M9	4.823	0.673
		M12	4.840	0.690	M15	4.808	0.658	M18	4.813	0.663
**5**	4.157	M3	4.426	0.269	M6	4.389	0.232	M9	4.388	0.231
		M12	4.518	0.361	M15	4.479	0.322	M18	4.493	0.336
**6**	4.604	M3	4.403	0.201	M6	4.373	0.231	M9	4.385	0.219
		M12	4.427	0.177	M15	4.398	0.206	M18	4.429	0.175
**7**	4.340	M3	4.532	0.192	M6	4.450	0.11	M9	4.501	0.161
		M12	4.635	0.295	M15	4.552	0.212	M18	4.613	0.273
**8**	4.324	M3	4.318	0.006	M6	4.290	0.034	M9	4.270	0.054
		M12	4.396	0.072	M15	4.364	0.040	M18	4.362	0.038

^1^ The experimental determination of the parameter pIC_50_ for compounds **1**–**8** is presented in Section 3. ^2^ΔpIC_50_ = pIC_50_^pred^ − pIC_50_^exp^.

**Table 6:** Numerical values of RMSEP and parameter 2·RMSER for models M3, M6, M9, M12, M15 and M18 estimated using Xternal Validation Plus 1.2 software based on TS1 and TS2.

Model	RMSEP				2·RMSEP			
	TS1		TS2		TS1		TS2	
	100% Data	95% Data	100% Data	95% Data	100% Data	95% Data	100% Data	95% Data
M3	0.437	0.389	-	-	0.874	0.778	-	-
M6	0.431	0.326	-	-	0.862	0.652	-	-
M9	0.440	0.375	-	-	0.880	0.750	-	-
M12	0.441	0.406	0.458	0.390	0.882	0.812	0.916	0.780
M15	0.425	0.365	0.420	0.362	0.850	0.730	0.840	0.724
M18	0.432	0.381	0.433	0.373	0.864	0.762	0.866	0.746

**Table 7:** Statistical characteristics of training sets TrS1–TrS2.

Designation of TrS_i_	Code of Training Set	
	TrS1	TrS2
N	84	70
$\bar{{pIC}_{50}}$	5.308	
∆pIC_50_	3.873	
Thresholds used to evaluate model’s forecast		
0.10 × ∆pIC_50_	0.387	
0.15 × ∆pIC_50_	0.581	
0.20 × ∆pIC_50_	0.775	
0.25 × ∆pIC_50_	0.968	

**Table 8:** Statistical characteristics of test sets TS1–TS2.

Designation of TS_i_	Code of Test Set	
	TS1	TS2
N	84	70
$\bar{{pIC}_{50}}$	4.765	4.678
∆pIC_50_	3.196	3.275
Distribution of the observed response values of test sets TSi around the test mean		
$\bar{{pIC}_{50}}$ ± 0.5, %	37.500	50.000
$\bar{{pIC}_{50}}$ ± 1.0, %	75.000	78.571
$\bar{{pIC}_{50}}\;\;$± 1.5, %	87.500	85.714
$\bar{{pIC}_{50\;}\;}$± 2.0, %	93.750	92.857
Distribution of the observed response values of test sets TSi around the training mean		
$\bar{{pIC}_{50}}$ ± 0.5, %	12.500	14.286
$\bar{{pIC}_{50}}$ ± 1.0, %	50.000	42.857
$\bar{{pIC}_{50}}\;\;$± 1.5, %	87.500	85.714
$\bar{{pIC}_{50\;\;}}$± 2.0, %	100.000	100.000

**Table 9:** Criteria for evaluating descriptive and predictive ability of QSPR M1–M18 models.

Model Quality	High Descriptive and Predictive Ability	Moderate Descriptive and Predictive Ability	Low Descriptive and Predictive Ability
$\mathrm{Criteria}\;\mathrm{based}\;\mathrm{on}\;R^{2}$	$R^{2}$$\to R_{0}^{2}$ > 0.8	$R^{2}$$\to R_{0}^{2}$ ≤ 0.8	$R^{2}$$\to R_{0}^{2}$ ≤ 0.6
	$\bar{{R^{2}}_{0}}$ > 0.8	$\bar{{R^{2}}_{0}}$ ≤ 0.6	$\bar{{R^{2}}_{0}}$ > 0.5
	$\bar{{R^{2}}_{m}}$ ≤ 0.15	$\bar{{R^{2}}_{m}}$ < 0.2	$\bar{{R^{2}}_{m}}$ < 0.2
	CCC > 0.8	CCC ≤ 0.8	CCC → 0.7
	$Q_{LMO}^{2}$ > 0.70	$Q_{LMO}^{2}$ ≤ 0.70	$Q_{LMO}^{2}$ < 0.60
	$Q_{F1}^{2}$ > 0.70	$Q_{F1}^{2}$ ≤ 0.70	$Q_{F1}^{2}$ < 0.60
	$Q_{F2}^{2}$ > 0.70	$Q_{F2}^{2}$ ≤ 0.70	$Q_{F2}^{2}$ < 0.60
	A * < 0.3	A * ≤ 0.3	A * > 0.3
MAE	MAE ≤ 0.387	MAE = (0.387; 0.581]	MAE > 0.581
Criteria B **	B ≤ 0.775	B = (0.775; 0.968]	B > 0.968

* The symbol of the parameter characterizing the stability of the model. It is calculated as the difference between the average values of the coefficients $R^{2}$ and $Q_{LMO}^{2}$ ($A=R^{2}-Q_{LMO}^{2}$). ** Criteria B are calculated based on the values MAE and SD (B=MAE+3SD).

## Data Availability

Data are contained within the article and Supplementary Materials.

## Supplementary Materials

The following supporting information can be downloaded at: https://www.mdpi.com/article/10.3390/molecules29235540/s1. Table S1. The equations for assessing the descriptive and predictive potentials of the QSAR models based on the R2 and MAE metrics; Table S2. The validation parameters of the QSAR models estimated using the Xternal Validation Plus 1.2 program based on the values of training set TrS1 from set S2 based on consensus models QSAR M1-M9; Table S3. The validation parameters of the QSAR models estimated using the Xternal Validation Plus 1.2 program based on the values of training set TrS2 from set S4 based on consensus models QSAR M10-M18; Table S4. The validation parameters of the QSAR models estimated using the Xternal Validation Plus 1.2 program based on the experimental and predicted values of TS1 from test set S3 based on consensus models QSAR M1-M9; Table S5. The validation parameters of the QSAR models estimated using the Xternal Validation Plus 1.2 program based on the experimental and predicted values of TS2 from test set S5 based on consensus models QSAR M10-M18; Table S6. The validation parameters of the QSAR models estimated using the Xternal Validation Plus 1.2 program based on the experimental and predicted values of TS1 from test set S3 based on consensus models QSAR M10-M18; Table S7. Statistical parameters and accuracy of the predicted pIC_50_ values of the compounds included in the training sets TrS1–TrS2 within the consensus models M1–M18; Table S8. The complete list of organic compounds from which the data set S1 was generated with their corresponding pIC50 characteristics.

## References

[1] Joo Y.C., Oh D.K. (2012). Lipoxygenases: Potential starting biocatalysts for the synthesis of signaling compounds. Biotechnol. Adv..

[2] Heshof R., de Graaff L.H., Villaverde J.J., Silvestre A.J.D., Haarmann T., Dalsgaard T.K., Buchert J. (2015). Industrial potential of lipoxygenases. Crit. Rev. Biotechnol..

[3] Hayward S., Cilliers T., Swart P. (2016). Lipoxygenases: From Isolation to Application. Compr. Rev. Food Sci. Food Saf..

[4] Haeggström J.Z., Funk C.D. (2011). Lipoxygenase and Leukotriene Pathways: Biochemistry, Biology, and Roles in Disease. Chem. Rev..

[5] Vincenti S., Mariani M., Alberti J.-C., Jacopini S., Brunini-Bronzini de Caraffa V., Berti L., Maury J. (2019). Biocatalytic Synthesis of Natural Green Leaf Volatiles Using the Lipoxygenase Metabolic Pathway. Catalysts.

[6] Mosblech A., Feussner I., Heilmann I. (2009). Oxylipins: Structurally diverse metabolites from fatty acid oxidation. Plant Physiol. Biochem..

[7] Upston J.M., Neuzil J., Witting P.K., Alleva R., Stocker R. (1997). Oxidation of free fatty acids in low density lipoprotein by 15-lipoxygenase stimulates nonenzymic, alpha-tocopherol-mediated peroxidation of cholesteryl esters. J. Biol. Chem..

[8] Mao F., Wu Y., Tang X., Wang J., Pan Z., Zhang P., Zhang B., Yan Y., Zhang X., Qian H. (2017). Human umbilical cord mesenchymal stem cells alleviate inflammatory bowel disease through the regulation of 15-LOX-1 in macrophages. Biotechnol. Lett..

[9] Vaezi M.A., Safizadeh B., Eghtedari A.R., Ghorbanhosseini S.S., Rastegar M., Salimi V., Tavakoli-Yaraki M. (2021). 15-Lipoxygenase and its metabolites in the pathogenesis of breast cancer: A double-edged sword. Lipids Health Dis..

[10] Hong S.H., Avis I., Vos M.D., Martínez A., Treston A.M., Mulshine J.L. (1999). Relationship of arachidonic acid metabolizing enzyme expression in epithelial cancer cell lines to the growth effect of selective biochemical inhibitors. Cancer Res..

[11] Orafaie A., Mousavian M., Orafai H., Sadeghian H. (2020). An overview of lipoxygenase inhibitors with approach of in vivo studies. Prostaglandins Other Lipid Mediat..

[12] Muñoz-Ramírez A., Mascayano-Collado C., Barriga A., Echeverría J., Urzúa A. (2020). Inhibition of Soybean 15-Lipoxygenase and Human 5-Lipoxygenase by Extracts of Leaves, Stem Bark, Phenols and Catechols Isolated From Lithraea caustica (Anacardiaceae). Front. Pharmacol..

[13] Wecksler A.T., Garcia N.K., Holman T.R. (2009). Substrate specificity effects of lipoxygenase products and inhibitors on soybean lipoxygenase-1. Bioorg. Med. Chem..

[14] Shaker B., Ahmad S., Lee J., Jung C., Na D. (2021). In silico methods and tools for drug discovery. Comput. Biol. Med..

[15] Chatterjee M., Roy K., Roy K. (2023). Chapter 1—Quantitative structure-activity relationships (QSARs) in medicinal chemistry. Cheminformatics, QSAR and Machine Learning Applications for Novel Drug Development.

[16] Tropsha A., Isayev O., Varnek A., Schneider G., Cherkasov A. (2024). Integrating QSAR modelling and deep learning in drug discovery: The emergence of deep QSAR. Nat. Rev. Drug Discov..

[17] Vijayalakshmi M.K., Srinivasan R. (2024). Review of Contemporary QSAR Study Approach. Chem. Afr..

[18] Ambure P., Gajewicz-Skretna A., Cordeiro M.N.D.S., Roy K. (2019). New Workflow for QSAR Model Development from Small Data Sets: Small Dataset Curator and Small Dataset Modeler. Integration of Data Curation, Exhaustive Double Cross-Validation, and a Set of Optimal Model Selection Techniques. J. Chem. Inf. Model..

[19] Verma J., Khedkar V.M., Coutinho E.C. (2010). 3D-QSAR in drug design—A review. Curr. Top. Med. Chem..

[20] Lagunin A.A., Romanova M.A., Zadorozhny A.D., Kurilenko N.S., Shilov B.V., Pogodin P.V., Ivanov S.M., Filimonov D.A., Poroikov V.V. (2018). Comparison of Quantitative and Qualitative (Q)SAR Models Created for the Prediction of Ki and IC50 Values of Antitarget Ingibitors. Front. Pharmacol..

[21] Filimonov D.A., Zakharov A.V., Lagunin A.A., Poroikov V.V. (2009). QNA based “Star Track” QSAR approach. SAR QSAR Environ. J. Res..

[22] Zakharov A.V., Peach M.L., Sitzmann M., Nicklaus M.C. (2014). A New Approach to Radial basis function approximation and Its application to QSAR. J. Chem. Inf. Model..

[23] Lagunin A.A., Geronikaki A., Eleftheriou P., Pogodin P.V. (2019). Rational Use of Heterogeneous Data in Quantitative Structure-Activity Relationship (QSAR) Modeling of Cyclooxygenase/Lipoxygenase Inhibitors. J. Chem. Inf. Model..

[24] Zakharov A.V., Peach M.L., Sitzmann M., Nicklaus M.C. (2014). QSAR modeling of imbalanced high-throughput screening data in PubChem. J. Chem. Inf. Model..

[25] Lagunin A., Zakharov A., Filimonov D., Poroikov V. (2011). QSAR Modelling of Rat Acute Toxicity on the Basis of PASS Prediction. Mol. Inform..

[26] Filimonov D.A., Akimov D.V., Poroikov V.V. (2004). The Method of Self-Consistent Regression for the Quantitative Analysis of Relationships Between Structure and Properties of Chemicals. Pharm. Chem. J..

[27] Ivanov S.M., Lagunin A.A., Filimonov D.A., Poroikov V.V. (2022). Relationships between the structure and severe drug-induced liver injury for low, medium, and high doses of drugs. Chem. Res. Toxicol..

[28] Lagunin A.A., Zakharov A.V., Filimonov D.A., Poroikov V.V. (2007). A new approach to QSAR modelling of acute toxicity. SAR QSAR Environ. Res..

[29] Zakharov A.V., Varlamova E.V., Lagunin A.A., Dmitriev A.V., Muratov E.N., Fourches D., Kuz’min V.E., Poroikov V.V., Tropsha A., Nicklaus M.C. (2016). QSAR Modeling and Prediction of Drug–Drug Interactions. Mol. Pharm..

[30] Martynova Y.Z., Khairullina V.R., Nasretdinova R.N., Garifullina G.G., Mitsukova D.S., Gerchikov A.Y., Mustafin A.G. (2020). Determination of the chain termination rate constants of the radical chain oxidation of organic compounds on antioxidant molecules by the QSPR method. Russ. Chem. Bull..

[31] Khairullina V., Safarova I., Sharipova G., Martynova Y., Gerchikov A. (2021). QSAR Assessing the Efficiency of Antioxidants in the Termination of Radical-Chain Oxidation Processes of Organic Compounds. Molecules.

[32] Khairullina V., Martynova Y., Safarova I., Sharipova G., Gerchikov A., Limantseva R., Savchenko R. (2022). QSPR Modeling and Experimental Determination of the Antioxidant Activity of Some Polycyclic Compounds in the Radical-Chain Oxidation Reaction of Organic Substrates. Molecules.

[33] Khairullina V.R., Martynova Y.Z. (2023). Quantitative Structure–Activity Relationship in the Series of 5-Ethyluridine, N2-Guanine, and 6-Oxopurine Derivatives with Pronounced Anti-Herpetic Activity. Molecules.

[34] Vinayagam J., Gajbhiye R.L., Mandal L., Arumugam M., Achari A., Jaisankar P. (2017). Substituted furans as potent lipoxygenase inhibitors: Synthesis, in vitro and molecular docking studies. Bioorganic Chem..

[35] Siskou I.C., Rekka E.A., Kourounakis A.P., Chrysselis M.C., Tsiakitzis K., Kourounakis P.N. (2007). Design and study of some novel ibuprofen derivatives with potential nootropic and neuroprotective properties. Bioorganic Med. Chem..

[36] Pontiki E., Hadjipavlou-Litina D. (2007). Synthesis and pharmacochemical evaluation of novel aryl-acetic acid inhibitors of lipoxygenase, antioxidants, and anti-inflammatory agents. Bioorganic Med. Chem..

[37] Wisastra R., Ghizzoni M., Boltjes A., Haisma H.J., Dekker F.J. (2012). Anacardic acid derived salicylates are inhibitors or activators of lipoxygenases. Bioorganic Med. Chem..

[38] Rao P.N.P., Chen Q.-H., Knaus E.E. (2005). Synthesis and biological evaluation of 1,3-diphenylprop-2-yn-1-ones as dual inhibitors of cyclooxygenases and lipoxygenases. Bioorganic Med. Chem. Lett..

[39] Doulgkeris C.M., Galanakis D., Kourounakis A.P., Tsiakitzis K.C., Gavalas A.M., Eleftheriou P.T., Victoratos P., Rekka E.A., Kourounakis P.N. (2006). Synthesis and pharmacochemical study of novel polyfunctional molecules combining anti-inflammatory, antioxidant, and hypocholesterolemic properties. Bioorganic Med. Chem. Lett..

[40] Burguete A., Pontiki E., Hadjipavlou-Litina D., Villar R., Vicente E., Solano B., Ancizu S., Pérez-Silanes S., Aldana I., Monge A. (2007). Synthesis and anti-inflammatory/antioxidant activities of some new ring substituted 3-phenyl-1-(1,4-di-N-oxide quinoxalin-2-yl)-2-propen-1-one derivatives and of their 4,5-dihydro-(1H)-pyrazole analogues. Bioorganic Med. Chem. Lett..

[41] Lau C.K., Belanger P.C., Scheigetz J., Dufresne C., Williams H.W.R., Maycock A.L., Guindon Y., Bach T., Dallob A.L. (1989). Synthesis and structure-activity relationships of a novel class of 5-lipoxygenase inhibitors. 2-(Phenylmethyl)-4-hydroxy-3,5-dialkylbenzofurans: The development of L-656,224. J. Med. Chem..

[42] Whitman S., Gezginci M., Timmermann B.N., Holman T.R. (2002). Structure−Activity Relationship Studies of Nordihydroguaiaretic Acid Inhibitors toward Soybean, 12-Human, and 15-Human Lipoxygenase. J. Med. Chem..

[43] Rao P.P.N., Chen Q.-H., Knaus E.E. (2006). Synthesis and Structure−Activity Relationship Studies of 1,3-Diarylprop-2-yn-1-ones: Dual Inhibitors of Cyclooxygenases and Lipoxygenases. J. Med. Chem..

[44] Kontogiorgis C.A., Hadjipavlou-Litina D.J. (2005). Synthesis and Antiinflammatory Activity of Coumarin Derivatives. J. Med. Chem..

[45] Shobha S.V., Candadai R.S., Ravindranath B. (1994). Inhibition of Soybean Lipoxygenase-1 by Anacardic Acids, Cardols, and Cardanols. J. Nat. Prod..

[46] Khan A.N., Perveen S., Malik A., Afza N., Iqbal L., Latif M., Saleem M. (2010). Conferin, potent antioxidant and anti-inflammatory isoflavone from *Caragana conferta* Benth. J. Enzym. Inhib. Med. Chem..

[47] Jabbari A., Sadeghian H., Salimi A., Mousavian M., Seyedi S.M., Bakavoli M. (2016). 2-Prenylated m-Dimethoxybenzenes as Potent Inhibitors of 15-Lipoxygenase: Inhibitory Mechanism and SAR studies. Chem. Biol. Drug Des..

[48] Rajić Z., Hadjipavlou-Litina D., Pontiki E., Balzarini J., Zorc B. (2011). The novel amidocarbamate derivatives of ketoprofen: Synthesis and biological activity. Med. Chem. Res..

[49] Doulgkeris C.M., Siskou I.C., Xanthopoulou N., Lagouri V., Kravaritou C., Eleftheriou P., Kourounakis P.N., Rekka E.A. (2012). Compounds against inflammation and oxidative insult as potential agents for neurodegenerative disorders. Med. Chem. Res..

[50] Dearden J.C., Cronin M.T.D., Kaiser K.L.E. (2009). How not to develop a quantitative structure-activity or structure-property relationship (QSAR/QSPR). SAR QSAR Environ. Res..

[51] Roy K., Kar S., Das R.N. (2015). Statistical Methods in QSAR/QSPR. A Primer on QSAR/QSPR Modeling.

[52] Gramatica P., Sangion A. (2016). A Historical Excursus on the Statistical Validation Parameters for QSAR Models: A Clarification Concerning Metrics and Terminology. J. Chem. Inf. Model..

[53] Xternal Validation Plus. https://sites.google.com/site/dtclabxvplus.

[54] MarvinSketch. https://chemaxon.com/download/marvin-suite.

[55] DiscoveryStudioVisualiser. https://www.3ds.com.

[56] Strashilina I.V. Zameshchennye 2n-piran-2-ony v one-pot sinteze n, o -soderzhashchikh geterosistem: avtoref. dis. … kand. khim. nauk. 02.00.03/Strashilina Irina Vladimirovna.–Saratov, 2018. –23 s. https://rusneb.ru/catalog/000199_000009_008707531/.

[57] Kanevskaya I.V., Bondartsova A.S., Fedotova O.V. (2020). Biginelli Synthesis of regioisomeric 5, 6-Dihydro-4 H-benzo[4,5]imidazo[1,2-a] pyranopyrimidin-4-ones. Russ. J. Org. Chem..

[58] Strashilina I.V., Arzyamova E.M., Fedotova O.V. (2018). Synthesis of fused 2 H-Pyridin-2-ones under the conditions of multicomponent Hantzsch reaction. Russ. J. Org. Chem..

[59] Lyckander I.M., Malterud K.E. (1992). Lipophilic flavonoids from Orthosiphon spicatus as inhibitors of 15-lipoxygenase. Acta Pharm. Nord..

[60] Malterud K.E., Rydland K.M. (2000). Inhibitors of 15-lipoxygenase from orange peel. J. Agric. Food Chem..

[61] Lyckander I.M., Malterud K.E. (1996). Lipophilic flavonoids from Orthosiphon spicatus prevent oxidative inactivation of 15-lipoxygenase. Prostaglandins Leukot. Essent. Fat. Acids.

