# Supplementary material for: QSAR Modeling and Biological Testing of Some 15-LOX Inhibitors in a Series of Homo- and Heterocyclic Compounds
Source: Molecules. 2024 Nov 23;29(23):5540. doi: 10.3390/molecules29235540 (PMC11643787; doi:10.3390/molecules29235540)
Supplement: Supplementary file 1 [file molecules-29-05540-s001.zip › molecules-3314941-supplementary.pdf]

Supplementary Material

CONTENTS

PARAMETERS FOR ASSESSING THE DESCRIPTIVE AND PREDICTIVE POTENTIAL OF QSAR MODELS 3

2. BRIEF DESCRIPTION OF THE GUSAR 2019 PROGRAM 6

2.1. CALCULATION OF STRUCTURAL DESCRIPTORS 6

2.2. SELECTION OF THE DESCRIPTORS WHEN CONSRTUCTING QSAR MODELS. 10

2.3. CONSTRUCTING QSAR MODELS 14

2.4. ASSESSMENT OF THE RANGE OF APPLICABILITY 15

3. RESULTS 16

PARAMETERS FOR ASSESSING THE DESCRIPTIVE AND PREDICTIVE POTENTIAL OF QSAR MODELS

Table S1. The equations for assessing the descriptive and predictive potentials of the QSAR models based on the R^2 and MAE metrics

Comment	Equation of criterion	
Parameters for assessing the descriptive and predictive potential of QSAR models using internal cross-validation techniques		
Determination coefficient (Coefficient of multiple determination R^2) is the determination coefficient of the calculated using the experimental and the predicted data of the training set	$R^2 = 1 - \frac{\sum_{i=1}^{N_{TrSi}}(y_i^{pred} - y_i^{obs})^2}{\sum_{i=1}^{N_{TrSi}}(y_i^{obs} - \overline{y^{obs}})^2} = 1 - \frac{RSS}{TSS};$ $R^2 = \left(\frac{\sum_{i=1}^{N_{TrSi}}(y_i^{obs} - \overline{y^{obs}})(y_i^{pred} - \overline{y^{pred}})}{\sqrt{\sum_{i=1}^{N_{TrSi}}(y_i^{obs} - \overline{y^{obs}})^2 \times \sum_{i=1}^{N_{TrSi}}(y_i^{pred} - \overline{y^{pre}}}} \right.$	(1)
R_0^2 and $R_0'^2$ are respectively the determination coefficients of the calculated using the experimental and the predicted data of the training set, forcing respectively the origin of the axis	$R_0^2 = 1 - \frac{\sum_{i=1}^{N_{TrSi}}\left(y_i^{pred} - k * y_i^{pred}\right)^2}{\sum_{i=1}^{N_{TrSi}}\left(y_i^{pred} - \overline{y^{pred}}\right)^2};$ $R'^2_0 = 1 - \frac{\sum_{i=1}^{N_{TrSi}}\left(y_i^{obs} - k' * y_i^{obs}\right)^2}{\sum_{i=1}^{N_{TrSi}}\left(y_i^{obs} - \overline{y^{obs}}\right)^2};$ $k = \frac{\sum_{i=1}^{N_{TrSi}}(y_i^{obs} * y_i^{pred})}{\sum_{i=1}^{N_{TrSi}}(y_i^{pred})^2}; k' = \frac{\sum_{i=1}^{N_{TrSi}}(y_i^{obs} * y_i^{pred})}{\sum_{i=1}^{N_{TrSi}}(y_i^{obs})^2}$	(2)
R^2_m is determination coefficient of the regression function, calculated using the experimental values on the ordinate axis, R'^2_m using them on the abscissa	$R_m^2 = R_{TrSi}^2\left(1 - \sqrt{R_{TrSi}^2 - R^2{}_{0\,TrSi}}\right) > 0.5;$ $\Delta R_m^2 = [R_m^2 - R'^2_m] < 0.2;$ $\overline{R_m^2} = \frac{R_m^2 + R'^2_m}{2}$	(3)

Determination coefficient by internal cross-validation	$Q^2 = Q^2_{20\%(n=20)} = 1 - \frac{\sum_{i=1}^{N_{TrSi}} \left(y_{i/i}^{pred} - y_i^{obs}\right)^2}{\sum_{i=1}^{N_{TrSi}} \left(y_i^{obs} - \overline{y^{obs}}\right)^2} = 1 - \frac{PRESS}{TSS}$	(4)
Standard deviation	$S.D. = \sqrt{\frac{\sum_{i=1}^{N_{TrSi}} \left(y_i^{obs} - y_i^{pred}\right)^2}{N_{TrSi} - V - 1}} = \sqrt{\frac{RSS}{N_{TrSi} - V - 1}}$	(5)
Root Mean Square Error in in prediction activity for training set	$RMSE = \sqrt{\frac{\sum_{i=1}^{N_{TrSi}} \left(y_i^{obs} - y_i^{pred}\right)^2}{N_{TrSi}}} = \sqrt{\frac{RSS}{N_{TrSi}}}$	(6)
Variance ratio (F)	$F = \frac{\sum_{i=1}^{N_{TrSi}} \left(y_i^{pred} - \overline{y^{obs}}\right)^2}{\sum_{i=1}^{N_{TrSi}} \left(y_i^{obs} - y_i^{pred}\right)^2} \times \frac{N_{TrSi} - V - 1}{V}$	(7)
Parameters for estimating the predictive power of QSAR models using external cross-validation technique		
R_0^2 and $R_0'^2$ are calculated forcing the regression line to pass through the origin, k and k' are the slope of the regression lines	$R_0^2 = 1 - \frac{\sum_{i=1}^{N_{TSi}} \left(y_i^{pred} - k * y_i^{pred}\right)^2}{\sum_{i=1}^{N_{TSi}} \left(y_i^{pred} - \overline{y^{pred}}\right)^2}$; $R_0'^2 = 1 - \frac{\sum_{i=1}^{N_{TSi}} \left(y_i^{obs} - k' * y_i^{obs}\right)^2}{\sum_{i=1}^{N_{TSi}} \left(y_i^{obs} - \overline{y^{obs}}\right)^2}$; $k = \frac{\sum_{i=1}^{N_{TSi}} \left(y_i^{obs} * y_i^{pred}\right)}{\sum_{i=1}^{N_{TSi}} \left(y_i^{pred}\right)^2}$; $k' = \frac{\sum_{i=1}^{N_{TSi}} \left(y_i^{obs} * y_i^{pred}\right)}{\sum_{i=1}^{N_{TSi}} \left(y_i^{obs}\right)^2}$	(8)

Correlation coefficient between observed and predicted activities	$R^2_{TSi} = 1 - \frac{\sum_{i=1}^{N_{TSi}}(y_i^{pred}-y_i^{obs})^2}{\sum_{i=1}^{N_{TSi}}(y_i^{obs}-\overline{y^{obs}})^2} = 1 - \frac{PRESS}{TSS};$ $R^2_{TSi} = \left(\frac{\sum_{i=1}^{N_{TSi}}(y_i^{obs}-\overline{y^{obs}})(y_i^{pred}-\overline{y^{pred}})}{\sqrt{\sum_{i=1}^{N_{TSi}}(y_i^{obs}-\overline{y^{obs}})^2 \times \sum_{i=1}^{N_{TSi}}(y_i^{pred}-\overline{y^{pred}})^2}}\right)^2$	(9)
The determination coefficients calculated for the connections of the test sample TS_i, taking into account the mean pIC_{50} for the training samples, the mean pIC_{50} for the test samples, respectively	$Q^2_{F_1} = 1 - \frac{\sum_{i=1}^{N_{TSi}}(y_i^{pred}-y_i^{obs})^2}{\sum_{i=1}^{N_{TSi}}\left(y_i^{obs}-\overline{y_{i/TrSi}^{obs}}\right)^2} = 1 - \frac{PRESS}{TSS_{test}(\overline{y_{i/TrSi}^{obs}})}$	(10)
	$Q^2_{F_2} = 1 - \frac{\sum_{i=1}^{N_{TSi}}(y_i^{pred}-y_i^{obs})^2}{\sum_{i=1}^{N_{TSi}}\left(y_i^{obs}-\overline{y_{i/TSi}^{obs}}\right)^2} = 1 - \frac{PRESS}{TSS_{test}\left(\overline{y_{i/TSi}^{obs}}\right)} = R^2_{TSi}$	(11)
Concordance Correlation Coefficient (CCC)	$CCC = \frac{2\sum_{i=1}^{N_{TSi}}(y_i^{obs}-\overline{y^{obs}})(y_i^{pred}-\overline{y^{pred}})}{\sum_{i=1}^{N_{TSi}}(y_i^{obs}-\overline{y^{obs}})^2 + \sum_{i=1}^{N_{TSi}}(y_i^{pred}-\overline{y^{pred}})^2 + N_{TSi}(\overline{y^{ob}}}$	(12)
R^2_m is determination coefficient of the regression function, calculated using the experimental values on the ordinate axis, R'^2_m using them on the abscissa	$R^2_m = R^2_{TSi}\left(1 - \sqrt{R^2_{TSi} - R^2_{0TSi}}\right) > 0.5;$ $\Delta R^2_m = [R^2_m - R'^2_m] < 0.2;$ $\overline{R^2_m} = \frac{R^2_m + R'^2_m}{2}$	(13)
Root Mean Square Error in prediction activity for test set	$RMSEP = \sqrt{\frac{\sum_{i=1}^{N_{TSi}}(y_i^{obs}-y_i^{pred})^2}{N_{TSi}}} = \sqrt{\frac{RSS}{N_{TSi}}}$	(14)
Mean Absolute Error	$MAE = \frac{\sum_{i=1}^{N_{TSi}}\lvert y_i^{obs}-y_i^{pred}\rvert}{N_{TSi}}$	(15)

where

TrSi is the training set, TSi is the test set,

N_{train} and N_{test} are total number of objects in the training set and test set respectively;

y_i^{obs} are experimental data values, y_i^{pred} are predicted data values,

$\overline{y^{obs}}$ are average of the experimental data values;

$\overline{y^{pred}}$ are average of the predicted data values;
RSS is residual sum of squares;
PRESS is the sum of the squares of the prediction errors (predictive sum of squares);
TSS is the total sum of squares (is sum of squared deviations from the data set mean); $TSS_{test}(\overline{y^{obs}_{i/train}})$ and $TSS_{test}(\overline{y^{obs}_{i/test}})$ are the total sum of squares of the external set calculated using the training set mean and external set mean, respectively.

2. BRIEF DESCRIPTION OF THE GUSAR 2019 PROGRAM
2.1. CALCULATION OF STRUCTURAL DESCRIPTORS

Here is a description of the GUSAR program necessary to understand the text of the article.

A detailed description of the ideology of calculating descriptors and constructing QSAR models using this program is given in the articles listed in the list of references and in the site http://www.pharmaexpert.ru (http://www.pharmaexpert.ru/passonline/downloads/articles/Filimonov-and-Poroikov-Chapter-6.pdf).

From a general point of view, the assessment of the activity of an organic molecule in the GUSAR2013 program is carried out according to the equation (1):

$$y_{\mathrm{pred}} = a_0 + \sum a_i f_i(S), \tag{1}$$

where a_0, a_1, … different functions of organic molecule's structure S.

In classic QSAR methods, the functions $f_1(S)$, $f_2(S)$, … represent physical-chemical parameters or other quantitative characteristics of molecular structure, and the coefficients a0, a1, ... are determined using multiple linear regression (MLR), partial least squares (PLS) analysis, or support vector regression (SVR), etc. QSAR methods based on the similarity between a certain molecule S_i with known biological activity and the molecule S use the value fiðSÞ of their similarity.

In the GUSAR 2019 program, the description of the structure and the calculation of the regression coefficients for the further construction of QSAR models is based on the use of two types of substructural descriptors of atomic neighborhoods: MNA (Multilevel Neighborhoods of Atoms) and QNA (Quantitative Neighborhoods of Atoms). They are automatically deduced from the matrices of molecular connectivity, standard ionization potentials (IP) and electron affinities (EA). The QNA descriptors are defined by two functions, P and Q. The P and Q values for each atom *i* are calculated using the following formulae [28-43]:

$$P_i = B_i \sum_k \left(\exp\left(\frac{-1}{2}C\right)\right)_{\mathrm{ik}} B_k \tag{2}$$

$$Q_i = B_i \sum_k \left(\exp\left(\frac{-1}{2}C\right)\right)_{\mathrm{ik}} B_k A_k \tag{3}$$

$$A_k = \frac{1}{2}(\mathrm{IP}_k + \mathrm{EA}_k), B_k = (\mathrm{IP}_k - \mathrm{EA}_k)^{-1/2} \tag{4}$$

where *k* is the remaining atoms in the molecule, IP is the first ionization potential, EA is the electron affinity for each atom (in eV), and C is the connectivity matrix for the molecule as a whole [28-39]. The standard values IP and EA of atoms in a molecule were collected from the literature. Although the value µP-Q can be considered by convention as the partial atomic

charge, where μ is the chemical potential, in general the P and Q values are not the estimate of partial atomic charges or hardness, etc.

Any atom influences the others, although the influence decreases with the increase of the distance between them. The algorithm of the QNA descriptor calculation is really very simple due to the uselessness of the matrix Exp(-1/2C) itself, the fact that the product of Exp(-1/2C) by a vectoris needed only, and the fact that the matrix C consists of 0 and 1 only. A detailed description of QNA descriptors is represented in [28-43].

Thus, the QNA descriptors are calculated taking into account the relationships between all atoms of the structure. These values describe each atom of the molecule but, at the same time, depend on the structure of the molecule as a whole.

In the future, based on the functions P and Q, the $f_i(S)$ functions are calculated. Each function of the structure of the molecule fi (S) is calculated according to equation (4) as the average value of the function gi (P, Q) for those m atoms of the molecule that have two or more immediate neighbors:

$$f_i(S) = \frac{1}{m}\sum g_i(P_k, Q_k) \quad (5)$$

Substitution of expression (5) into equation (1) and permutation of the sums allows one to obtain equation (6):

$$y_{\text{pred}} = a_0 + \sum a_i \frac{1}{m}\sum g_i(P_k, Q_k) = \frac{1}{m}\sum(a_0 + \sum a_i\, g_i(P_k, Q_k)) \quad (6)$$

Thus, in accordance with equation (6), the estimate of the parameter y_{pred} for a molecule is the average of the predicted values for specific atoms in the molecule. Formally, QNA descriptors represent the structure of a molecule with only two descriptors (P and Q), in contrast to the many traditional descriptors used in QSAR.

However, the developers of the GUSAR program found that the P and Q values are highly correlated with each other (r = 0.903). Since the values of P and Q have different scales (standard deviations are 0.023 and 0.208, respectively), the developers of the GUSAR program carried out normalization to optimize the family of functions g_i (P, Q). Normalization was performed by calculating mean values (E_P and E_Q), standard deviations (D_P and D_Q), and correlation between P and Q values (R_{PQ}):

$$P' = \frac{P - E_p}{D_p} \qquad Q' = \frac{Q - E_Q}{D_Q} \quad (7)$$

$$u = \frac{P' + Q'}{\sqrt{2(1 + R_{\text{PQ}})}}\ u = \frac{P' - Q'}{\sqrt{2(1 - R_{\text{PQ}})}} \quad (8)$$

The orthonormal U and V have zero mean, unit variance, and they are uncorrelated [28-39].

The QNA values are the basic information for calculating the Chebyshev 2D polynomials.

$$g_i(P,Q) = T_{\mathrm{uv}}(P,Q) = \cos(u \cdot \arccos(\mathrm{TanH}(u))) \cdot \cos(v \cdot \arccos(\mathrm{TANH}(v))) \qquad (9)$$

where the integers u, v=0, 1, 2, ... define the 2D Chebyshev polynomial degree. The final equation for estimate y_{pred} using QNA descriptors is

$$y_{\mathrm{pred}} = \frac{1}{m}\sum(a_0 + \sum a_{\mathrm{uv}} T_{\mathrm{uv}}(P_k, Q_k)) = a_0 + \sum a_{\mathrm{uv}} T_{\mathrm{uv}}$$
$$T_{\mathrm{uv}} = \frac{1}{m}\sum(T_{\mathrm{uv}}(P_k, Q_k) \qquad (10)$$

Thus, the regression equations constructed in the GUSAR 2019 program take into account both the specificity and physicochemical properties of each atom entering the training set [28-39]. However, QNA descriptors cannot be physically interpreted due to the peculiarities of their calculation. In this regard, they are not explicitly displayed under calculations.

The MNA descriptors are computed using the PASS algorithm (Prediction of Activity Spectra for Substances), which predicts approximately 6,400 "biological activities" with an accuracy threshold of an average prediction of at least 95%. These descriptors are generated based on the structural formulae of chemical compounds without using any pre-compiled list of structural fragments [28-39]. The authors of the GUSAR 2019 program report that "MNA-descriptors are based on the molecular structure representation, which includes hydrogens according to the valences and partial charges of other atoms and does not specify the types of bonds." They are generated as "a recursively defined sequence:

• zero-level MNA descriptor for each atom is the mark A of the atom itself;

• any next-level MNA descriptor for the atom is the substructure notation A (D1D2…Di…), where Di is the previous-level MNA descriptor for i–th immediate neighbor of the atom A.

The neighbor descriptors $D_1D_2 \ldots D_i \ldots$ are arranged in a unique manner. This may be, for example, a lexicographic sequence. MNA descriptors are generated using an iterative procedure, which results in the formation of structural descriptors that include the first, second, etc. neighborhoods of each atom. The label contains not only information about the type of atom, but also additional information about its belonging to a cyclic or acyclic system, etc. For example, an atom that does not enter a ring is marked with a "—".

Based on the MNA descriptors using B-statistics, calculated in the PASS program, the biological activity spectrum of a chemical compound is predicted.

The output of the PASS program is the probabilities of the activity (Pa) of inactivity (Pi) of each prognostic result. The difference between these two values (Pa–Pi) for a randomly

selected subset of predicted activities is used as independent variables for regression analysis in GUSAR. GUSAR 2019 incorporates a PASS version that pedicts 4130 types of biological activity. The developers of the GUSAR 2019 program report that the list of predictable biological activities currently includes 501 pharmacotherapeutic effects, 3295 mechanisms of action, 57 adverse and toxic effects, 199 metabolic terms, 49 transporter proteins and 29 activities related to gene expression. The average accuracy of a reliable prediction of biological activity, calculated by leave-one-out cross-validation procedure is approximately 95% [30-39].

However, the regression equation constructed based on the MNA descriptors reveals the specificity of the action of the compound but does not explicitly reflects the physicochemical parameters of chemical compounds.

In addition, the GUSAR 2019 program calculates the QSAR descriptors of an entire molecule such as topological length, topological volume, lipophilicity, and physicochemical descriptors (numbers of positive and negative charges, number of donors and acceptors of the hydrogen bond, number of aromatic atoms, molecular weight and number of halogen atoms). Therefore, these parameters were added to the QNA descriptors. The topological length of a molecule was calculated as the maximal distance between any two atoms and the volume of a molecule as the sum of each atom's volume, $4/3\pi R^3$, where R is the atomic radius.

The authors of the GUSAR 2019 program report that "in GUSAR, the scale of QNA- and PASS-based descriptors ranges from −1 to 1. Therefore, no additional normalization is required for these types of descriptors. Only whole-molecule descriptors are normalized using a standard Z-score normalization procedure" [35-39].

It should be noted that the program is able to construct QSAR models both relying solely on one of these types of descriptors, and on their combination in terms of the consensus approach. At the same time, based on the consensus approach methodology, models for quantitative prediction of biological activity for these descriptors are calculated independently of each other. The examples of the sample QSAR GUSAR models for predicting the toxic effects of chemical compounds are available free *via* the link http://www.way2drug.com/GUSAR.

However, it noteworthy that the features of the QNA and MNA calculations retain these descriptors without unambiguous physical interpretation. For this reason, in the commercial and academic versions of the GUSAR 2019 program for broad use, the regression equations are not displayed.

2.2. SELECTION OF THE DESCRIPTORS WHEN CONSRTUCTING QSAR MODELS

In GUSAR 2019, three approaches are used when selecting the optimal number of descriptors for constructing QSAR-models:

1) self-consistent regression method (SCF) [36];

2) method of radial basis functions (RBF) [31];

3) method based on the combination of SCF and RBF [31, 39].

The SCF and SCF-RBF methods are the most preferable. The SCF method is correctly applied to modeling compounds with a rather high degree of similarity. The other two methods of selecting the optimal number of descriptors show good results when modeling structurally dissimilar compounds.

It is obvious that any regressor has only a restricted influence on the response, i.e. large values of regression coefficients are prohibitive, i.e. they have small probabilities. We therefore suggest using an a priori probability distribution of the regression coefficients p(a|v), where v are the distribution parameters. Therefore, the estimate of a is obtained by the maximum a posteriori probability method

$$a = \text{argmax} p(a \vee X\text{,y,v}) \quad (11)$$

where p(a|***X, y, v***) is calculated by Bayes formula:

$$p(a \vee \text{X,y, v}) = \frac{p(y \vee X\text{,a})p(a \vee v)}{p(y \vee X\text{,v})} \quad (12)$$

and the likelihood function of the sample **p(y|X,v)** is calculated by summation(integration) of all possible values of the regression coefficients **a:**

$$p(y \vee \text{X, v}) = \sum_a p(y \vee X\text{,a})p(a \vee v) \quad (13)$$

If the residuals $\boldsymbol{\varepsilon} = \boldsymbol{y} - \boldsymbol{Xa}$ are normally distributed and an a priori conditional probability ***p(a|v)*** has also normal density:

$$p(a \vee v) \sim \exp\left[\frac{-(v_1 a_1^2 + \ldots + v_m a_m^2)}{2}\right] \quad (14)$$

It was previously shown [28-39] that self-consistent regression (SCR) can be successfully applied to various QSAR problems. The SCR method is resistant to noise in the data and allows deleting the variables that poorly describe the target value. This is a regularized method of the least squares. Independent parameters *a* are calculated in this method according to the equation (15) [39]:

$$a = \text{ArgMin}[(\sum_{i=1}^{n} y_i - \sum_{k=0}^{m} x_{\text{ik}} a_k)^2 + \sum_{k=1}^{m} v_k a_k^2] \quad (15)$$

where *a* is the regression coefficient, *n* is the number of objects, y_i is the response value of the *i*-th object, *m* is the number of independent variables, x_{ik} is the value of the *k*-th independent

variable of the i-th object, a_k is the k-th value of the regression coefficients, and v_k is the k-th value of the regularization parameters. Equation (15) has the following solution:

$$a = \mathrm{T}\mathrm{X}^T y, \mathrm{Var}(a) = \sigma^2 T, T = (X^T X + \sigma^2 V)^{-1} \quad (16)$$

where X^T is the transposed regression matrix X, and $\sigma^2 V$ is the diagonal matrix of the regularization parameters. The regression coefficients obtained from the SCR reflect the contribution of each particular descriptor (variable) to the final equation. The higher the absolute value of the coefficient, the greater its contribution. Thus, the regression coefficients obtained after the SCR can be used to weight the descriptors (variables) depending on their importance.

Since the parameters v use the same data sample, X and y, we called the method ''self-consistent regression'' (SCR). As recommended in [33-39], the maximum likelihood method can be used to find the best values of parameters v:

$$a = \mathrm{argmax} p(y \vee X, \mathrm{v}) \quad (17)$$

In cases where *p(**y**|**X**,**a**)* and *p(**a**|**v**)* are the normal densities from equations (13)–(17), the following equation is derived:

$$v_k(a_k^2 + a^2 t_k) = 1, k = 1,..., m, \quad (18)$$

where t_k is the kth diagonal element of matrix T.

Due to their complex multidimensional nonlinear character, equation (18) can only be solved by iteration methods. Unlike the stepwise regression and other methods of combinatorial search, the SCR model includes all regressors. Nevertheless, the final model may contain several regressors truly describing the existing relationship. They can be easily identified based on their significance, which can be presented by the effective dimension of the regressor:

$$d_k(1 - \sigma^2 v_k t_k) = 1, k = 1,..., m, \quad (19)$$

Only those meeting a certain criterion, e.g. $d_k > 10^{-2}$, are left in the model.

The assumption of normality for *p(**y**|**X**,**a**)* and *p(**a**|**v**)* is not as restricted as seems to be the case. Normal distribution has an extreme property: it has the highest entropy for distributions with equal dispersion, and, in this sense, it is the ''worst'' among all possible distributions. Therefore, a solution obtained under the assumption of normality is rougher than it is theoretically possible for an exact residual distribution, but it is more robust, which is essential for the predictive power of a regression model. The regularized least-squares method (6) can be applied directly, without any statistical paradigm. However, the above-discussed statistical approach offers a useful tool for the optimisation of parameters v.

If residuals' dispersion σ^2 is unknown, then the following estimate s 2 can be used:

$$s^2 = \frac{\sum_i y_i(y_i - \sum_k x_{ik} a_k)}{(\mathrm{n\text{-}d})}, d = 1 + \sum_k d_k \quad (20)$$

Based on the above-described theory, we have developed an efficient SCR algorithm.

It is based on a modified Gram–Schmidt orthogonalization, which does not require the explicit inversion of a high-dimension matrix [31].

For a test molecule, the value of y can be calculated as follows:

$$y = \sum_k x_k a_k \,, k = 1,\ldots,m \tag{21}$$

where m is the number of regressors (qQNA) left in the equation after the SCR-part of the training procedure.

The second method used implemented in the GUSAR 2019 program for selecting the optimal number of descriptors is the interpolation method for radial basis functions RBF [31]. The authors of the GUSAR 2019 program reports [31] that, unlike the RBF network, this method uses each input variable as a center of gravity. The learning process is performed on all input variables of the training set. As can be seen from equation (22), the approximating function y(x) in the case of the RBF interpolation is represented as the sum of *N* radial basis functions, each of which is related to another center x_i and weighted by the corresponding coefficient w_i.

$$y(x) = \sum_{i=1}^{N} w_i \, \phi(\|x - x_i\|) = \Phi w \tag{22}$$

If the points x_i are different then the interpolation matrix Φ in the above equation is nonsingular. The weights w are calculated as:

$$w = \Phi^{-1} y \tag{23}$$

Assessing the weights is based on the simple least squares method [31, 39].

The RBF-SCR method is the third tool of the GUSAR 2019 program for selecting the optimal number of descriptors. It has a 3-step algorithm:

1) selecting descriptors using the SCF method;
2) calculating the radial basis functions using the weighted coefficient of SCR as a criterion of similarity;
3) calculating the weighting coefficients RBF by the least squares.

The RBF-SCR method can be expressed as [31]:

$$y(x) = \sum_{i=1}^{N} w_i \, \phi(\|\mathrm{ax} - a_i x_i\|) = \Phi w, \tag{24}$$

where a is taken from equation (15). Weights a_i are a new elements as compared to equation (22).

The RBF and RBF-SCR interpolation is based on a linear radial basis function that allows modeling a variety of training sets with a high level of dissimilarity between the objects.

Additionally, the GUSAR program allows visualizing the contribution of each atom into the predicted value [36-43]. This capability is implemented in the QSAR models based on the

QNA descriptors and, accordingly, in the consensus combination of the QSAR models designed in different modes. It opens opportunities to identify “strong” and “weak” points in the biologically active molecules and, consequently, to rationalize the conclusions about the replacement of certain fragments upon molecular design directed to enhancing/weakening the target property.

2.3. CONSTRUCTING QSAR MODELS

The QSAR models were designed in the GUSAR 2019 program as follows. To describe the structures of compounds within the program, two types of atom-centered descriptors were used, *viz.* substructural MNA, electrotopological QNA, and, additionally, three descriptors of the whole molecule (topological length, topological volume, and lipophilicity).

Self-consistent regression was used as a mathematical algorithm [36]. Previously, it has been shown [33] that self-consistent regression (SCR) can be successfully used to generate models from a large number of descriptors under different noise levels in the data. This method is correctly applied to modeling compounds with a rather high degree of similarity. Two other methods of selecting the optimal number of descriptors demonstrate good results when searching for quantitative structure–activity relationships in a series of structurally dissimilar compounds. As the TYMS inhibitors are structurally similar, the RBF and RBF-SCF methods were not used in the present work.

The descriptors were automatically calculated from the structural formulas of chemical compounds, taking into account the valence and partial charges of the atoms. The optimal set of the descriptors for constructing particular regression equations was automatically selected by the self-consistent regression and sliding control procedures [32, 36, 38]. The GUSAR 2019 program allows constructing both private regression dependencies and consensus models based on them. In this study, we use the consensus approach to construct the QSAR models. This allows reducing the variability of the predictions. Consensus models were designed in GUSAR 2019 automatically based on the principle of common similarity of particular regression dependencies [38-39].

Note that each of these partial models involved by the consensus model was made independently based on either QNA or MNA descriptors. As a result, 12 consensus QSAR models were designed. These models included 360 partial models. However, not all of them had acceptable statistical parameters. To select the most predictive models, a 20-fold crosscheck was performed for each model. These models have the R^2 values exceed 0.6 (from the cross-validation procedure after the randomized rejection of 20% of the training set). Each of the final

consensus models M1–M2, M4–M5, M7–M8, M10–M11 is made up with 20 particular regression dependencies. Consensus models M3, M6, M9 and M12 include 320 regression equations. However, as the QNA and MNA descriptors have no direct physical meaning, the regression equations constructed on their basis are not explicitly displayed in the GUSAR 2019 program. Only the QSAR models satisfying the abovementioned condition have been further used for numerical predicting pIC_{50} for the compounds of the external training set.

2.4. ASSESSMENT OF THE RANGE OF APPLICABILITY

To assess the applicability of models, GUSAR 2019 provides three different approaches based on similarity, leverage, and accuracy previously described in detail [31, 39-43].

Similarity. Using the Pearson correlation coefficients for each compound, we calculated the distances toward its nearest neighbors in the training set in the space of independent variables obtained after SCR. The compound is considered in the range of the model's applicability if the average value of these three distances is lower or equal to 0.7.

Leverage. The calculation of leverage allows estimating the contribution of each molecule to its own predicted value:

$$\text{Leverage} = x^T(X^TX)^{-1}x, \qquad (25)$$

where x is the vector of descriptors of the tested compound and **X** is the matrix made up with rows corresponding to the descriptors of all the molecules of the training set. The compound is considered out of the applicability range if its leverage is larger than 99 % in the distribution of the leverage values of the training set.

Accuracy degree (AD). Here, the prediction of the applicability range for each compound is calculated based on the prediction error for the three most similar compounds in the test set relative to the training set as a whole:

$$\text{AD}_{\text{value}} = \text{RMSE}_{\text{3NN}}/\text{RMSE}_{\text{train}} \qquad (26)$$

In the present study, a threshold value of 1 was used for AD.

3. RESULTS

Table S2.The validation parameters of the QSAR models estimated using the Xternal Validation Plus 1.2 program based on the values of training set TrS1 from set S2 based on consensus models QSAR M1-M9;

Comments	Prediction parameters	QSAR model used for predicting pIC50								
		TS1								
		SCR			RBF-SCR			Both		
		M1	M2	M3	M4	M5	M6	M7	M8	M9
Classical Metrics (100% data)	R^2	0.936	0.932	0.950	0.990	0.984	0.988	0.985	0.980	0.986
	R^2_0	0.928	0.920	0.937	0.989	0.984	0.987	0.981	0.976	0.982
	$R^{2'}_0$	0.907	0.891	0.915	0.989	0.983	0.986	0.979	0.972	0.979
	$\overline{R^2_m}$	0.959	0.953	0.963	0.995	0.992	0.993	0.990	0.987	0.990
	ΔR^2_m	0.060	0.067	0.053	0.009	0.014	0.011	0.015	0.020	0.015
	CCC	0.959	0.953	0.963	0.995	0.992	0.993	0.990	0.987	0.990
Classical Metrics (after removing 5% data with highresiduals)	R^2	0.942	0.942	0.956	0.993	0.992	0.992	0.986	0.985	0.989
	R^2_0	0.937	0.933	0.946	0.992	0.991	0.991	0.984	0.982	0.986
	$R^{2'}_0$	0.812	0.786	0.805	0.963	0.950	0.956	0.920	0.912	0.919
	$\overline{R^2_m}$	0.856	0.837	0.849	0.971	0.964	0.965	0.934	0.927	0.933
	ΔR^2_m	0.053	0.057	0.046	0.006	0.007	0.007	0.014	0.015	0.012
	CCC	0.965	0.962	0.970	0.996	0.995	0.995	0.991	0.990	0.992
Mean absolute errorand standarddeviation for test set (100% data)	RMSE	0.263	0.278	0.247	0.101	0.126	0.113	0.134	0.152	0.133
	MAE	0.216	0.225	0.205	0.079	0.089	0.086	0.110	0.121	0.110
	SD	0.151	0.165	0.139	0.063	0.089	0.073	0.076	0.094	0.076
	MAE+3·SD	0.151	0.165	0.139	0.063	0.089	0.073	0.076	0.094	0.076
Mean absolute errorand standarddeviation for test set (after removing 5% data with highresiduals)	RMSE	0.237	0.244	0.220	0.088	0.094	0.094	0.121	0.128	0.116
	MAE	0.237	0.244	0.220	0.088	0.094	0.094	0.121	0.128	0.116
	SD	0.134	0.140	0.119	0.053	0.059	0.056	0.068	0.072	0.062
	MAE+3·SD	0.134	0.140	0.119	0.053	0.059	0.056	0.068	0.072	0.062
Distribution ofprediction errors (in %)	Nω in range $0.10\cdot\Delta pIC_{50\ (TrS1)}$	13.1	16.67	13.1	0	1.19	1.19	0	1.19	1.19
	Nω in range $0.15\cdot\Delta pIC_{50\ (TrS1)}$	1.19	3.57	0	0	0	0	0	0	0
	Nω in range $0.20\cdot\Delta pIC_{50\ (TrS1)}$	0	0	0	0	0	0	0	0	0
	Nω in range $0.25\cdot\Delta pIC_{50\ (TrS1)}$	0	0	0	0	0	0	0	0	0
Prediction quality	-	Good								
Systematic errorpresence	-	Absent								

Table S3.The validation parameters of the QSAR models estimated using the Xternal Validation Plus 1.2 program based on the values of training set TrS2 from set S4 based on consensus models QSAR M10-M18;

Comments	Prediction parameters	QSAR model used for predicting pIC50								
		TS1								
		SCR			RBF-SCR			Both		
		M10	M11	M12	M13	M14	M15	M16	M17	M18
Classical Metrics (100% data)	R^2	0.923	0.937	0.949	0.986	0.983	0.986	0.982	0.980	0.985
	R^2_0	0.914	0.925	0.934	0.985	0.983	0.985	0.977	0.976	0.981
	$R^{2'}_0$	0.886	0.899	0.910	0.984	0.981	0.984	0.974	0.972	0.977
	$\overline{R^2_m}$	0.813	0.818	0.821	0.959	0.953	0.949	0.916	0.913	0.915
	ΔR^2_m	0.071	0.063	0.056	0.012	0.014	0.012	0.018	0.020	0.016
	CCC	0.951	0.956	0.962	0.992	0.991	0.992	0.988	0.987	0.990
Classical Metrics (after removing 5% data with highresiduals)	R^2	0.934	0.944	0.953	0.990	0.991	0.991	0.984	0.986	0.989
	R^2_0	0.927	0.935	0.942	0.990	0.990	0.990	0.981	0.983	0.985
	$R^{2'}_0$	0.781	0.787	0.790	0.955	0.948	0.948	0.907	0.914	0.912
	$\overline{R^2_m}$	0.832	0.835	0.834	0.969	0.965	0.964	0.923	0.929	0.927
	ΔR^2_m	0.062	0.055	0.051	0.008	0.008	0.008	0.016	0.014	0.012
	CCC	0.959	0.964	0.967	0.995	0.994	0.995	0.990	0.991	0.992
Mean absolute errorand standarddeviation for test set (100% data)	RMSE	0.290	0.271	0.254	0.120	0.130	0.121	0.149	0.153	0.138
	MAE	0.240	0.214	0.211	0.092	0.094	0.092	0.123	0.118	0.113
	SD	0.164	0.168	0.144	0.078	0.091	0.078	0.084	0.097	0.080
	MAE+3·SD	0.733	0.717	0.642	0.326	0.368	0.327	0.376	0.409	0.353
Mean absolute errorand standarddeviation for test set (after removing 5% data with highresiduals)	RMSE	0.260	0.242	0.228	0.103	0.101	0.101	0.134	0.130	0.119
	MAE	0.260	0.242	0.228	0.103	0.101	0.101	0.134	0.130	0.119
	SD	0.142	0.147	0.125	0.064	0.064	0.060	0.074	0.077	0.064
	MAE+3·SD	0.142	0.147	0.125	0.064	0.064	0.060	0.074	0.077	0.064
Distribution ofprediction errors (in %)	Nω in range 0.10·ΔpIC_{50} (TrS1)	20	17.14	14.29	0	1.43	1.43	0	1.43	1.43
	Nω in range 0.15·ΔpIC_{50} (TrS1)	2.86	2.86	0	0	0	0	0	0	0
	Nω in range 0.20·ΔpIC_{50} (TrS1)	0	0	0	0	0	0	0	0	0
	Nω in range 0.25·ΔpIC_{50} (TrS1)	0	0	0	0	0	0	0	0	0
Prediction quality	-	Good								
Systematic errorpresence	-	Absent								

Table S4.The validation parameters of the QSAR models estimated using the Xternal Validation Plus 1.2 program based on the experimental and predicted values of TS1 from test set S3 based on consensus models QSAR M1-M9; ΔpIC_{50} (TrS1) = 3.8725; ΔpIC_{50} (TS1) = 3.1963

Comments	Prediction parameters	QSAR model used for predicting pIC50								
		TS1								
		SCR			RBF-SCR			Both		
		M1	M2	M3	M4	M5	M6	M7	M8	M9
Classical Metrics (100% data)	R^2	0.8211	0.7847	0.7782	0.8091	0.7866	0.7839	0.7995	0.7943	0.7757
	R^2_0	0.8175	0.7831	0.7777	0.8084	0.7852	0.7838	0.7991	0.7940	0.7756
	$R^{2'}_0$	0.8053	0.7482	0.7300	0.7780	0.7506	0.7304	0.7603	0.7533	0.7109
	Q^2_{F1}	0.8640	0.8381	0.8345	0.8573	0.8398	0.8390	0.8494	0.8460	0.8327
	Q^2_{F2}	0.8174	0.7826	0.7777	0.8083	0.7849	0.7838	0.7978	0.7932	0.7753
	$\overline{R^2_m}$	0.7614	0.7133	0.7047	0.7451	0.7160	0.7112	0.7329	0.7245	0.7003
	ΔR^2_m	0.0403	0.1048	0.1167	0.0826	0.0996	0.1326	0.0794	0.1320	0.1432
	CCC	0.9056	0.8832	0.8776	0.8966	0.8844	0.8795	0.8890	0.8875	0.8727
Classical Metrics (after removing 5% data with highresiduals)	R^2	0.8452	0.8217	0.7982	0.8492	0.8554	0.8272	0.8233	0.8415	0.8070
	R^2_0	0.8279	0.8102	0.7898	0.8405	0.8458	0.8223	0.8158	0.8345	0.8033
	$R^{2'}_0$	0.8201	0.7585	0.7034	0.7967	0.8125	0.7375	0.7442	0.7747	0.6938
	Q^2_{F1}	0.8894	0.8751	0.8664	0.8982	0.8966	0.8859	0.8856	0.8888	0.8758
	Q^2_{F2}	0.8203	0.7971	0.7829	0.8346	0.8319	0.8146	0.8141	0.8194	0.7983
	$\overline{R^2_m}$	0.7932	0.7621	0.7318	0.7979	0.8057	0.7683	0.7646	0.7868	0.7422
	ΔR^2_m	0.0157	0.0542	0.0683	0.0284	0.0490	0.0844	0.0272	0.0831	0.0962
	CCC	0.9159	0.9022	0.8915	0.9196	0.9198	0.9070	0.9070	0.9121	0.8963
Mean absolute errorand standarddeviation for test set (100% data)	RMSEP	0.3963	0.4324	0.4372	0.4060	0.4301	0.4312	0.4171	0.4217	0.4396
	MAE	0.3432	0.3607	0.3767	0.3550	0.3434	0.3696	0.3561	0.3519	0.3724
	SD	0.2045	0.2463	0.2292	0.2034	0.2675	0.2295	0.2242	0.2400	0.2411
	MAE+3·SD	0.9568	1.0997	1.0643	0.9651	1.1458	1.0580	1.0287	1.0720	1.0958
Mean absolute errorand standarddeviation for test set (after removing 5% data with highresiduals)	RMSEP	0.3541	0.3762	0.3892	0.3397	0.3424	0.3597	0.3601	0.3550	0.3751
	MAE	0.3131	0.3226	0.3427	0.3152	0.2932	0.3264	0.3188	0.3102	0.3313
	SD	0.1711	0.2004	0.1909	0.1309	0.1830	0.1564	0.1733	0.1786	0.1823
	MAE+3·SD	0.8264	0.9238	0.9154	0.7081	0.8423	0.7955	0.8386	0.8460	0.8780
Distribution ofprediction errors (in %)	Nω in range 0.10·ΔpIC_{50} (TrS1)	37.5000	43.7500	50.0000	37.5000	43.7500	43.7500	37.5000	37.5000	43.7500
	Nω in range 0.15·ΔpIC_{50} (TrS1)	12.5000	18.7500	12.5000	6.2500	6.2500	6.2500	12.5000	12.5000	6.2500
	Nω in range 0.20·ΔpIC_{50} (TrS1)	6.2500	6.2500	6.2500	6.2500	6.2500	6.2500	6.2500	6.2500	6.2500
	Nω in range 0.25·ΔpIC_{50} (TrS1)	0.0000	0.0000	0.0000	0.0000	6.2500	6.2500	0.0000	6.2500	6.2500
Prediction quality		Moderate			Good	Moderate				
Systematic errorpresence		Absent								

Table S5.The validation parameters of the QSAR models estimated using the Xternal Validation Plus 1.2 program based on the experimental and predicted values of TS2 from test set S5 based on consensus models QSAR M10-M18; ΔpIC_{50} (TrS2) = 3.8725; ΔpIC_{50} (TS2) = 3.2747

Comments	Prediction parameters	QSAR model used for predicting pIC50								
		TS2								
		SCR			RBF-SCR			Both		
		M10	M11	M12	M13	M14	M15	M16	M17	M18
Classical Metrics (100% data)	R^2	0.8101	0.7227	0.7661	0.8488	0.7649	0.8043	0.8325	0.7497	0.7916
	R^2_0	0.8094	0.7211	0.7607	0.8478	0.7619	0.7977	0.8315	0.7473	0.7843
	$R^{2\prime}_0$	0.7528	0.5798	0.6315	0.8092	0.6495	0.6989	0.7846	0.6255	0.6706
	Q^2_{F1}	0.8651	0.8048	0.8347	0.8936	0.8347	0.8609	0.8833	0.8231	0.8517
	Q^2_{F2}	0.8037	0.7159	0.7594	0.8452	0.7595	0.7976	0.8301	0.7426	0.7842
	$\overline{R^2_m}$	0.6940	0.5743	0.6121	0.7482	0.6219	0.6626	0.7321	0.6013	0.6424
	ΔR^2_m	0.1451	0.2036	0.1828	0.1176	0.1794	0.1563	0.1277	0.1896	0.1664
	CCC	0.8906	0.8310	0.8546	0.9146	0.8576	0.8788	0.9051	0.8479	0.8695
Classical Metrics (after removing 5% data with highresiduals)	R^2	0.7936	0.7303	0.7666	0.8802	0.7678	0.7896	0.8012	0.7423	0.7772
	R^2_0	0.7843	0.7224	0.7644	0.8677	0.7630	0.7885	0.7946	0.7362	0.7762
	$R^{2\prime}_0$	0.6994	0.5831	0.6095	0.6436	0.6311	0.6375	0.7001	0.5949	0.6138
	Q^2_{F1}	0.8784	0.8434	0.8733	0.9142	0.8699	0.8910	0.8928	0.8528	0.8842
	Q^2_{F2}	0.7464	0.6734	0.7356	0.8676	0.7286	0.7725	0.7764	0.6929	0.7585
	$\overline{R^2_m}$	0.7204	0.6330	0.6634	0.7423	0.6766	0.6964	0.7310	0.6445	0.6769
	ΔR^2_m	0.1338	0.1864	0.1693	0.1041	0.1656	0.1541	0.1131	0.1806	0.1628
	CCC	0.8773	0.8364	0.8626	0.9213	0.8628	0.8803	0.8887	0.8450	0.8720
Mean absolute errorand standarddeviation for test set (100% данные)	RMSEP	0.4134	0.4972	0.4576	0.3670	0.4575	0.4197	0.3845	0.4733	0.4334
	MAE	0.3405	0.4112	0.3736	0.3137	0.3644	0.3371	0.3298	0.3881	0.3552
	SD	0.2432	0.2901	0.2742	0.1977	0.2872	0.2594	0.2051	0.2812	0.2577
	MAE+3·SD	1.0702	1.2815	1.1962	0.9068	1.2258	1.1154	0.9452	1.2316	1.1283
Mean absolute errorand standarddeviation for test set (after removing 5%data with highresiduals)	RMSEP	0.3819	0.4334	0.3899	0.3417	0.3951	0.3617	0.3586	0.4202	0.3726
	MAE	0.3125	0.3651	0.3271	0.2912	0.3194	0.2958	0.3066	0.3474	0.3127
	SD	0.2285	0.2429	0.2208	0.1861	0.2420	0.2167	0.1935	0.2461	0.2110
	MAE+3·SD	0.9978	1.0939	0.9895	0.8495	1.0455	0.9457	0.8871	1.0856	0.9457
Distribution ofprediction errors (in %)	Nω in range $0.10 \cdot \Delta pIC_{50}$ (TrS2)	35.7143	50.0000	50.0000	42.8571	50.0000	50.0000	42.8571	42.8571	42.8571
	Nω in range $0.15 \cdot \Delta pIC_{50}$ (TrS2)	21.4286	21.4286	14.2857	14.2857	21.4286	14.2857	14.2857	21.4286	14.2857
	Nω in range $0.20 \cdot \Delta pIC_{50}$ (TrS2)	0.0000	14.2857	7.1429	0.0000	7.1429	7.1429	0.0000	14.2857	7.1429
	Nω in range $0.25 \cdot \Delta pIC_{50}$ (TrS2)	0.0000	7.1429	7.1429	0.0000	0.0000	0.0000	0.0000	0.0000	0.0000
Prediction quality		Bad			Moderate	Bad	Moderate		Bad	Moderate
Systematic errorpresence		Absent								

Table S6.The validation parameters of the QSAR models estimated using the Xternal Validation Plus 1.2 program based on the experimental and predicted values of TS1 from test set S3 based on consensus models QSAR M10-M18; ΔpIC_{50} (TrS2) = 3.8725; ΔpIC_{50} (TS1) = 3.1963

Comments	Prediction parameters	QSAR model used for predicting pIC50								
		TS1								
		SCR			RBF-SCR			Both		
		M10	M11	M12	M13	M14	M15	M16	M17	M18
Classical Metrics (100% data)	R^2	0.8180	0.7921	0.7757	0.8180	0.8321	0.7903	0.8145	0.8262	0.7835
	R^2_0	0.8163	0.7911	0.7750	0.8179	0.8319	0.7902	0.8145	0.8261	0.7835
	R'^2_0	0.7955	0.7553	0.7282	0.7835	0.8055	0.7435	0.7711	0.7880	0.7229
	Q^2_{F1}	0.8615	0.8406	0.8320	0.8641	0.8722	0.8437	0.8618	0.8667	0.8388
	Q^2_{F2}	0.8141	0.7860	0.7744	0.8174	0.8283	0.7900	0.8145	0.8209	0.7835
	$\overline{R^2_m}$	0.7558	0.7175	0.7009	0.7537	0.7612	0.7193	0.7449	0.7319	0.7077
	ΔR^2_m	0.1007	0.1426	0.1312	0.1264	0.1193	0.1340	0.1293	0.1274	0.1474
	CCC	0.9027	0.8859	0.8767	0.9009	0.9088	0.8844	0.8973	0.9028	0.8779
Classical Metrics (after removing 5% data with highresiduals)	R^2	0.8702	0.8420	0.8222	0.8542	0.8616	0.8120	0.8476	0.8587	0.8021
	R^2_0	0.8695	0.8409	0.8209	0.8486	0.8611	0.8055	0.8442	0.8563	0.7982
	R'^2_0	0.7256	0.6649	0.6269	0.7908	0.7108	0.7188	0.7648	0.6790	0.6856
	Q^2_{F1}	0.9026	0.8818	0.8657	0.8994	0.8968	0.8745	0.8992	0.8930	0.8719
	Q^2_{F2}	0.8689	0.8409	0.8192	0.8366	0.8610	0.7961	0.8362	0.8560	0.7918
	$\overline{R^2_m}$	0.8153	0.7599	0.7507	0.8035	0.7906	0.7488	0.7946	0.7615	0.7356
	ΔR^2_m	0.0922	0.1134	0.1230	0.0779	0.0997	0.0860	0.0896	0.1064	0.1052
	CCC	0.9278	0.9107	0.8965	0.9200	0.9236	0.8981	0.9179	0.9190	0.8930
Mean absolute errorand standarddeviation for test set (100% data)	RMSEP	0.3999	0.4290	0.4405	0.3963	0.3842	0.4249	0.3995	0.3924	0.4315
	MAE	0.3330	0.3711	0.3747	0.3263	0.3290	0.3653	0.3267	0.3437	0.3639
	SD	0.2287	0.2224	0.2391	0.2321	0.2049	0.2243	0.2375	0.1955	0.2396
	MAE+3·SD	1.0192	1.0382	1.0920	1.0227	0.9439	1.0381	1.0391	0.9302	1.0826
Mean absolute errorand standarddeviation for test set (after removing 5% data with highresiduals)	RMSEP	0.3457	0.3809	0.4061	0.3377	0.3560	0.3771	0.3380	0.3624	0.3811
	MAE	0.2968	0.3373	0.3468	0.2884	0.3057	0.3317	0.2874	0.3198	0.3285
	SD	0.1835	0.1830	0.2187	0.1818	0.1888	0.1859	0.1842	0.1764	0.2000
	MAE+3·SD	0.8474	0.8864	1.0029	0.8338	0.8722	0.8892	0.8400	0.8490	0.9283
Distribution ofprediction errors (in %)	Nω in range 0.10·$\Delta pIC_{50 \, (TrS2)}$	31.2500	50.0000	50.0000	37.5000	37.5000	50.0000	31.2500	43.7500	50.0000
	Nω in range 0.15·$\Delta pIC_{50 \, (TrS2)}$	12.5000	12.5000	18.7500	12.5000	12.5000	12.5000	12.5000	12.5000	18.7500
	Nω in range 0.20·$\Delta pIC_{50 \, (TrS2)}$	6.2500	6.2500	12.5000	6.2500	0.0000	6.2500	6.2500	0.0000	6.2500
	Nω in range 0.25·$\Delta pIC_{50 \, (TrS2)}$	0.0000	0.0000	0.0000	0.0000	0.0000	0.0000	0.0000	0.0000	0.0000
Prediction quality	-	Moderate		Bad	Moderate					
Systematic errorpresence	-	Absent								

Table S7. Statistical parameters and accuracy of the predicted pIC_{50} values of the compounds included in the training sets TrS1–TrS2 within the consensus models M1–M18. pIC_{50} TrS1 = pIC50 TrS2 = 3.873, pIC50 TS1 = 3.196; pIC50 TS2 = 3.2751.

	Метод	TrSi	Модель	N	N_{mod}	R^2_{OBi}	Q^2_{OBi}	F	SD	R^2_{TBi}	V
based on the QNA descriptors	SCR	TrS1	M1	84	20	0.825	0.758	10.429	0.485	0.821	17
		TrS2	M10	70	20	0.804	0.714	7.608	0.531	0.818*<br>0.810**	15
	RBF-SCR	TrS1	M4	84	20	0.997	0.802	14.606	0.437	0.809	17
		TrS2	M13	70	20	0.996	0.753	10.204	0.492	0.818*<br>0.849**	15
	Both	TrS1	M7	84	20	0.962	0.800	13.026	0.443	0.800	17
		TrS2	M16	70	20	0.959	0.757	9.264	0.491	0.815*<br>0.833**	15
based on the MNA descriptors	SCR	TrS1	M2	84	20	0.798	0.725	8.749	0.517	0.785	16
		TrS2	M11	70	20	0.825	0.741	6.444	0.512	0.792*<br>0.723**	17
	RBF-SCR	TrS1	M5	84	20	0.985	0.745	11.115	0.495	0.787	16
		TrS2	M14	70	20	0.982	0.725	7.267	0.518	0.832*<br>0.765**	17
	Both	TrS1	M8	84	20	0.955	0.760	10.365	0.486	0.794	16
		TrS2	M17	70	20	0.959	0.756	7.170	0.495	0.826*<br>0.750**	17
Based on both (QNA &MNA) descriptors	SCR	TrS1	M3	84	320	0.842	0.777	8.747	0.480	0.778	17
		TrS2	M12	70	320	0.842	0.766	7.067	0.499	0.776*<br>0.766**	16
	RBF-SCR	TrS1	M6	84	320	0.991	0.783	11.373	0.460	0.784	17
		TrS2	M15	70	320	0.990	0.769	9.189	0.480	0.790*<br>0.804**	16
	Both	TrS1	M16	84	320	0.965	0.798	10.443	0.454	0.776	17
		TrS2	M108	70	320	0.966	0.787	8.401	0.474	0.784*<br>0.792**	16

To verify the validity of these models, we used:

* — an external TrS1 test sample;

** — internal TrS2 test sample.

Table S8. The complete list of organic compounds from which the data set S1 was generated with their corresponding pIC50 characteristics

Structure	BindingDB Ligand Name	IC50 (nM)
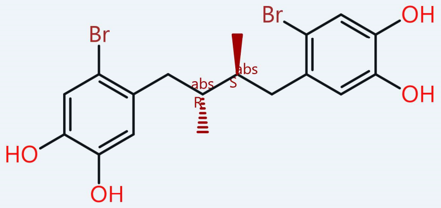	4-bromo-5-[(2R,3S)-4-(2-bromo-4,5-dihydroxyphenyl)-2,3-dimethylbutyl]benzene-1,2-diol::CHEMBL89283	57
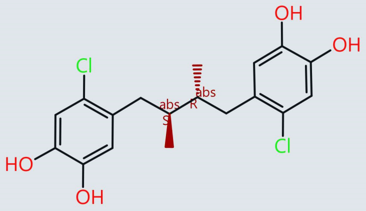	4-chloro-5-[(2R,3S)-4-(2-chloro-4,5-dihydroxyphenyl)-2,3-dimethylbutyl]benzene-1,2-diol::CHEMBL314941	65
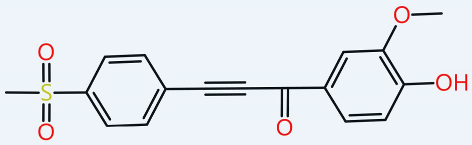	1,3-Diarylprop-2-yn-1-one, 23a::1-(4-hydroxy-3-methoxyphenyl)-3-(4-methanesulfonylphenyl)prop-2-yn-1-one::CHEMBL372548	100
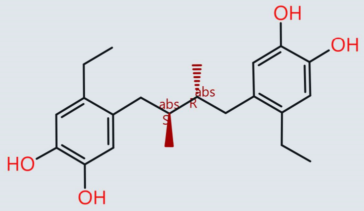	4-ethyl-5-[4-(2-ethyl-4,5-dihydroxyphenyl)-2,3-dimethyl-(2S,3R)-butyl]-1,2-benzenediol::CHEMBL92212	140
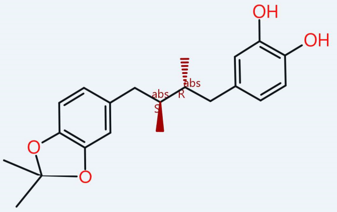	4-[4-(2,2-dimethylbenzo[d][1,3]dioxol-5-yl)-2,3-dimethyl-(2R,3S)-butyl]-1,2-benzenediol::CHEMBL88952	140
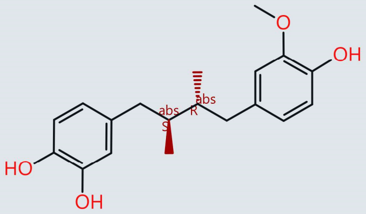	4-[(2S,3R)-4-(4-Hydroxy-3-methoxy-phenyl)-2,3-dimethyl-butyl]-benzene-1,2-diol::4-[4-(4-Hydroxy-3-methoxy-phenyl)-2,3-dimethyl-butyl]-benzene-1,2-diol::CHEMBL316139	170
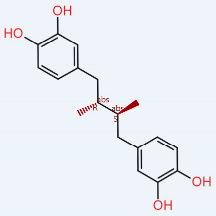	4-[(2R,3S)-4-(3,4-dihydroxyphenyl)-2,3-dimethylbutyl]benzene-1,2-diol::4-[(2S,3R)-3-[(3,4-dihydroxyphenyl)methyl]-2-methylbutyl]benzene-1,2-diol::CHEMBL313972::Masoprocol::NDGA::NordihydroguaiareticAcid	180
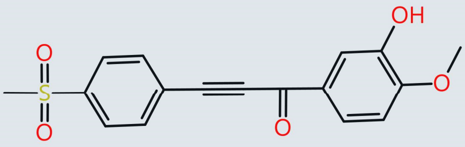	1,3-Diarylprop-2-yn-1-one, 23b::1-(3-hydroxy-4-methoxyphenyl)-3-(4-methanesulfonylphenyl)prop-2-yn-1-one::CHEMBL196084	300

Structure	BindingDB Ligand Name	IC50 (nM)
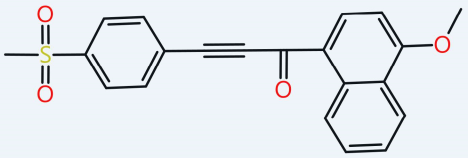	1,3-Diarylprop-2-yn-1-one, 17c::3-(4-methanesulfonylphenyl)-1-(4-methoxynaphthalen-1-yl)prop-2-yn-1-one	300
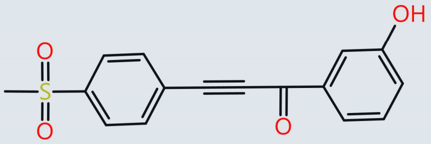	1,3-Diarylprop-2-yn-1-one, 13m::1-(3-hydroxyphenyl)-3-(4-methanesulfonylphenyl)prop-2-yn-1-one::CHEMBL364739	300
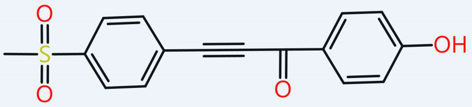	1,3-Diarylprop-2-yn-1-one, 13k::1-(4-hydroxyphenyl)-3-(4-methanesulfonylphenyl)prop-2-yn-1-one::CHEMBL194297	300
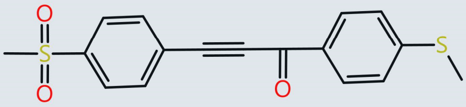	1,3-Diarylprop-2-yn-1-one, 27::3-(4-methanesulfonylphenyl)-1-[4-(methylsulfanyl)phenyl]prop-2-yn-1-one	360
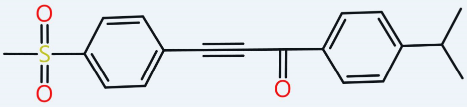	1,3-Diarylprop-2-yn-1-one, 13e::3-(4-methanesulfonylphenyl)-1-[4-(propan-2-yl)phenyl]prop-2-yn-1-one	360
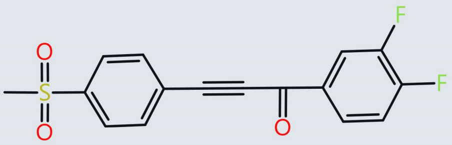	1,3-Diarylprop-2-yn-1-one, 13i::1-(3,4-difluorophenyl)-3-(4-methanesulfonylphenyl)prop-2-yn-1-one::CHEMBL197573	400
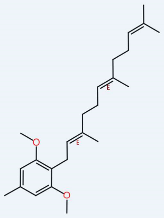	1,3-Dimethoxy-5-methyl-2-((2E,6E)-3,7,11-trimethyldodeca-2,6,10-trien-1-yl)benzene(11)	520
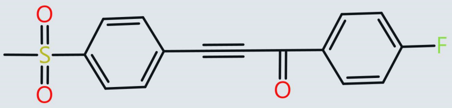	1,3-Diarylprop-2-yn-1-one, 13h::1-(4-fluorophenyl)-3-(4-methanesulfonylphenyl)prop-2-yn-1-one::CHEMBL427356	1000

Structure	BindingDB Ligand Name	IC50 (nM)
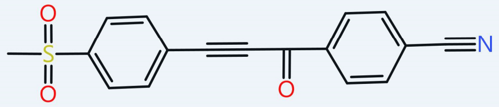	1,3-Diarylprop-2-yn-1-one, 13g::4-[3-(4-methanesulfonylphenyl)prop-2-ynoyl]benzonitrile::CHEMBL362923	1000
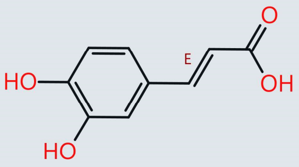	(2E)-3-(3,4-dihydroxyphenyl)prop-2-enoic acid::(2E)-3-(3,4-dihydroxyphenyl)prop-2-enoicacid (9)::3,4-Dihydroxycinnamate,XVII::CHEMBL145::Caffeic acid::Caffeic acid, 1::cid_689043	3000
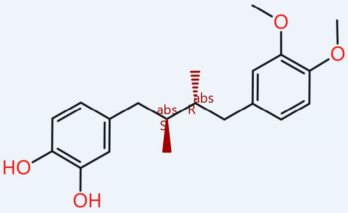	4-[(2S,3R)-4-(3,4-Dimethoxy-phenyl)-2,3-dimethyl-butyl]-benzene-1,2-diol::4-[4-(3,4-Dimethoxy-phenyl)-2,3-dimethyl-butyl]-benzene-1,2-diol::CHEMBL91531	3100
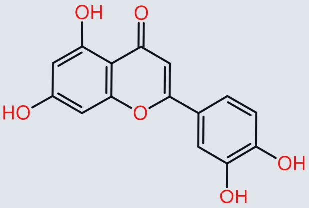	2-(3,4-dihydroxyphenyl)-5,7-dihydroxy-4H-chromen-4-one::2-(3,4-dihydroxyphenyl)-5,7-dihydroxy-chromen-4-one::CHEMBL151::Luteolin(27)::Luteolin(4)::cid_5280445::luteolin	3200
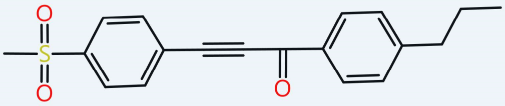	1,3-Diarylprop-2-yn-1-one, 13c::3-(4-methanesulfonylphenyl)-1-(4-propylphenyl)prop-2-yn-1-one	3200
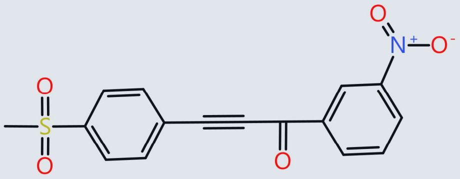	1,3-Diarylprop-2-yn-1-one, 26c::3-(4-methanesulfonylphenyl)-1-(3-nitrophenyl)prop-2-yn-1-one	3500
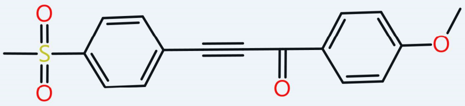	1,3-Diarylprop-2-yn-1-one, 13j::3-(4-methanesulfonylphenyl)-1-(4-methoxyphenyl)prop-2-yn-1-one::CHEMBL371191	3500
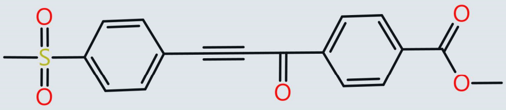	1,3-Diarylprop-2-yn-1-one,13p::methyl 4-[3-(4-methanesulfonylphenyl)prop-2-ynoyl]benzoate	5000

Structure	BindingDB Ligand Name	IC50 (nM)
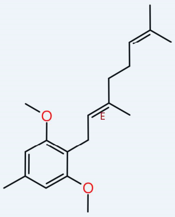	(E)-2-(3,7-Dimethylocta-2,6-dien-1-yl)-1,3-dimethoxy-5-methylbenzene (10)	5300
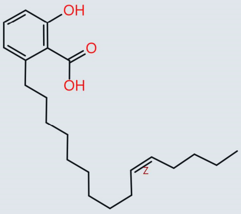	Anacardicacid10'Z-monoene::CHEMBL470264	6000
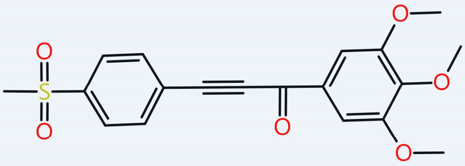	1,3-Diarylprop-2-yn-1-one, 13o::3-(4-methanesulfonylphenyl)-1-(3,4,5-trimethoxyphenyl)prop-2-yn-1-one::CHEMBL194891	7000
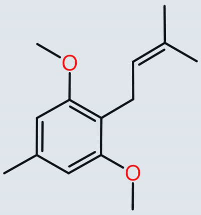	1,3-Dimethoxy-5-methyl-2-(3-methylbut-2-en-1-yl)benzene (9)	7600
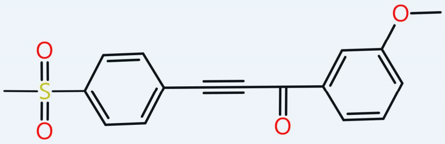	1,3-Diarylprop-2-yn-1-one, 13l::3-(4-methanesulfonylphenyl)-1-(3-methoxyphenyl)prop-2-yn-1-one::CHEMBL194298	9000
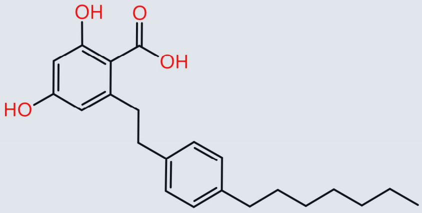	CHEMBL1934604	11100
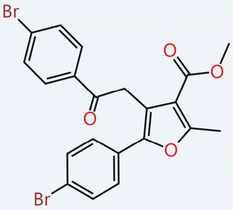	5-(4-Bromo-phenyl)-4[2-(4-bromo-phenyl)-2-oxo-ethyl]-2-methyl-furan-3-carboxylic acid methyl ester (3e)	12800
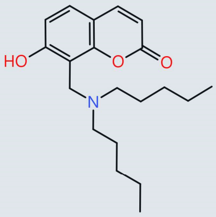	8-Dipentylaminomethyl-7-hydroxy-chromen-2-one::CHEMBL197190	13000

Structure	BindingDB Ligand Name	IC50 (nM)
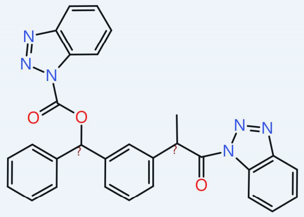	CHEMBL1172493	21000
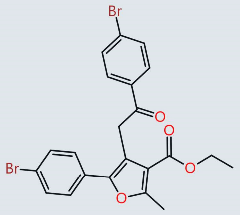	5-(4-Bromo-phenyl)-4[2-(4-bromo-phenyl)-2-oxo-ethyl]-2-methyl-furan-3-carboxylic acid ethyl ester (3f)	21200
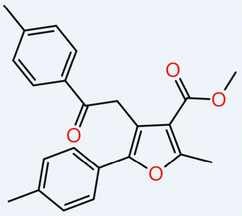	Methyl 2-methyl-5-(4-methylphenyl)-4-[2-(4-methylphenyl)-2-oxoethyl]furan-3-carboxylate(3c)	22100
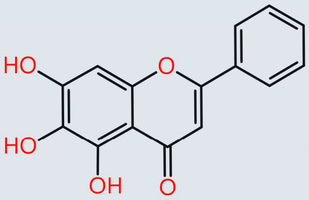	5,6,7-Trihydroxyflavone::5,6,7-trihydroxy-2-phenyl-4H-chromen-4-one::Baicalein::Baicalein,14::Baicalein, 8::CHEMBL8260::US10252984,Table2.33	22600
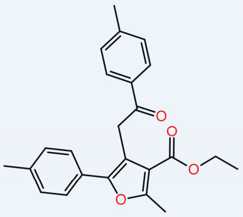	2-Methyl-4-(2-oxo-2-p-tolyl-ethyl)-5-p-tolyl-furan-3-carboxylic acid ethyl ester (3d)	22600
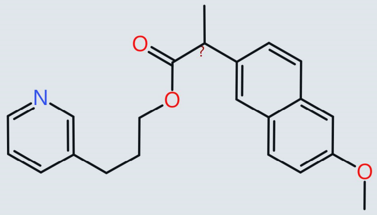	CHEMBL2262178	28000
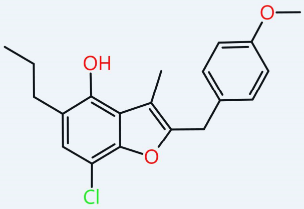	7-Chloro-2-(4-methoxy-benzyl)-3-methyl-5-propyl-benzofuran-4-ol::CHEMBL17815	30000
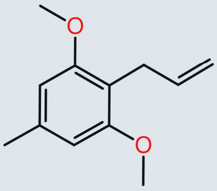	2-Allyl-1,3-dimethoxy-5-methylbenzene(8)	30500

Structure	BindingDB Ligand Name	IC50 (nM)
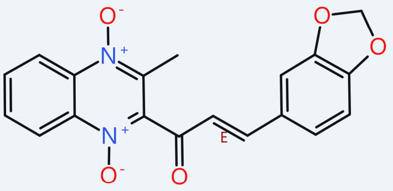	(E)-3-benzo[1,3]dioxol-5-yl-1-(3-methyl-1,4-dioxy-quinoxalin-2-yl)-propenone::2-[3-(3,4-Methylenedioxyphenyl)-2-propenoyl]-3-methyl-quinoxaline-1,4-dioxide::CHEMBL237949	31000
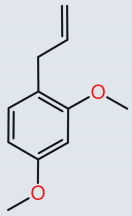	1-Allyl-2,4-dimethoxybenzene (4)::1-Allyl-2,4-dimethoxybenzene(5)	31400
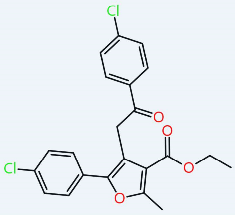	5-(4-Chloro-phenyl)-4[2-(4-chloro-phenyl)-2-oxo-ethyl]-2-methyl-furan-3-carboxylic acid ethyl ester (3h)	32000
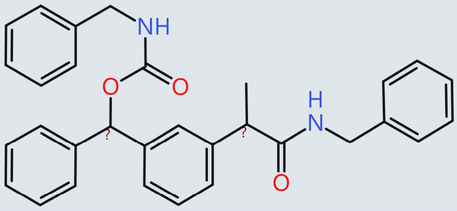	(3-(1-(Benzylcarbamoil)Ethyl)Phenyl)(Phenyl)Methylbenzyl carbamate::CHEMBL1170394	32000
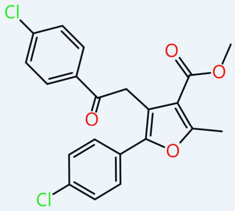	5-(4-Chloro-phenyl)-4[2-(4-chloro-phenyl)-2-oxo-ethyl]-2-methyl-furan-3-carboxylic acid methyl ester (3g)	32500
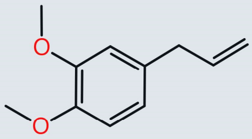	METHYLEUGENOL::Methyleugenol(1)	33300
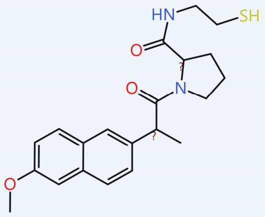	CHEMBL204551	35000
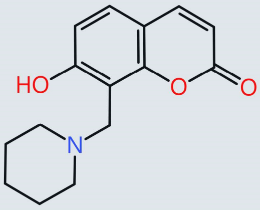	7-Hydroxy-8-piperidin-1-ylmethyl-chromen-2-one::8-piperidinomethyl-7-hydroxycoumarin::CHEMBL193416	37000

Structure	BindingDB Ligand Name	IC50 (nM)
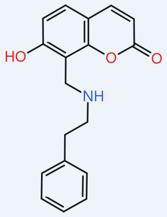	7-Hydroxy-8-(phenethylamino-methyl)-chromen-2-one::CHEMBL196156	41000
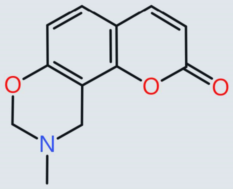	3-Methyl-3,4-dihydro-2H-1,5-dioxa-3-aza-phenanthren-6-one::CHEMBL195020	42000
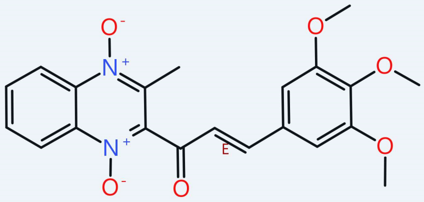	(E)-1-(3-methyl-1,4-dioxy-quinoxalin-2-yl)-3-(3,4,5-trimethoxy-phenyl)-propenone::3-Methyl-2-[3-(3,4,5-trimethoxyphenyl)-2-propenoyl]-quinoxaline-1,4-dioxide::CHEMBL237950	42000
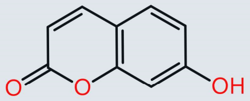	7-hydroxy-2H-1-benzopyran-2-one::7-hydroxy-2H-chromen-2-one::CHEMBL51628::Coumarin derivative, 3a::beta-umbelliferone::hydrangin::skimmetin::umbelliferone	43000
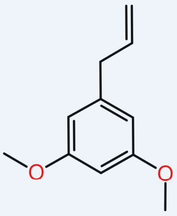	1-Allyl-3,5-dimethoxybenzene(2)	43200
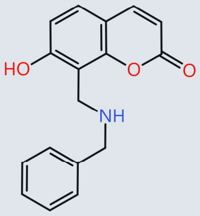	8-(Benzylamino-methyl)-7-hydroxy-chromen-2-one::CHEMBL196052	44000
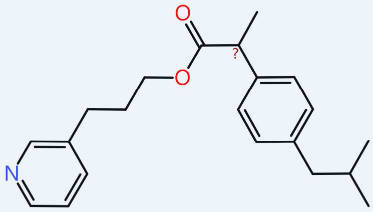	CHEMBL2262185	45000
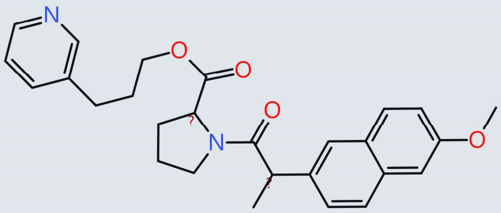	CHEMBL2262370	46000

Structure	BindingDB Ligand Name	IC50 (nM)
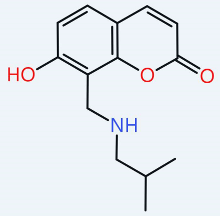	7-Hydroxy-8-(isobutylamino-methyl)-chromen-2-one::CHEMBL194961	47000
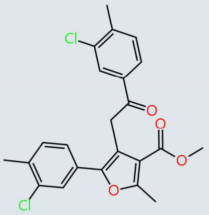	5-(3-Chloro-4-methyl-phenyl)-4-[2-(3-chloro-4-methyl-phenyl)-2-oxo-ethyl]-2-methyl-furan-3-carboxylic acid methyl ester (3i)	47500
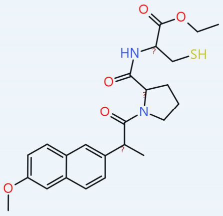	CHEMBL436935	48000
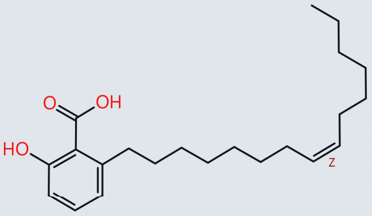	6-(8'Z-pentadecenyl)-salicylicacid::6-[8'(Z)-pentadecenyl]salicylicacid::6-[8(Z)-pentadecatrienyl]salicylic acid::6-{8(Z)-pentadecenyl}salicylicacid::6[8'(Z)-pentadecenyl]salicylic acid::Anacardic acid 8'Z-monoene::CHEMBL445177::Ginkgolic acid C15:1 (1)	50000
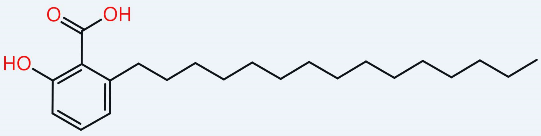	2-Hydroxy-6-pentadecyl-benzoic acid::2-hydroxy-6-pentadecylbenzoic acid::6-(pentadecenyl)salicylic acid::anacardic acid	51900
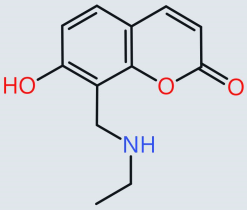	8-Ethylaminomethyl-7-hydroxy-chromen-2-one::CHEMBL196527	52000
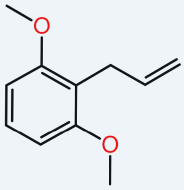	2-Allyl-1,3-dimethoxybenzene(6)	53200
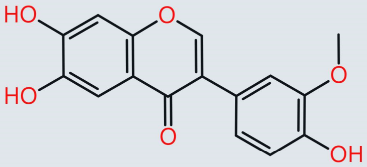	Conferin (1)	55200

Structure	BindingDB Ligand Name	IC50 (nM)
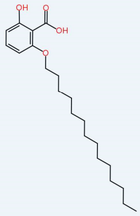	CHEMBL418973	55400
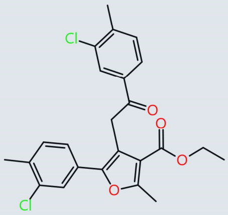	5-(3-Chloro-4-methyl-phenyl)-4-[2-(3-chloro-4-methyl-phenyl)-2-oxo-ethyl)-2-methyl-furan-3-carboxylic acid methyl ester (3j)	56500
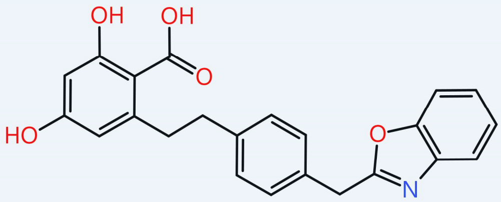	CHEMBL2206368	58500
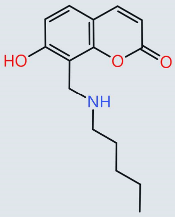	7-Hydroxy-8-pentylaminomethyl-chromen-2-one::CHEMBL194450	59000
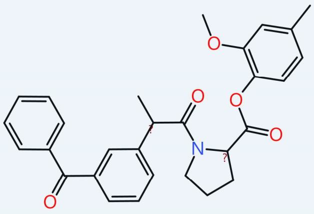	CHEMBL2262368	60000
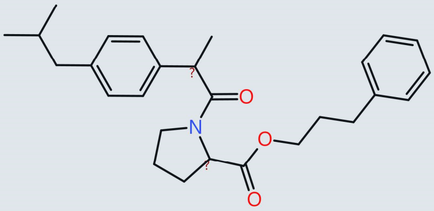	CHEMBL2262180	60000
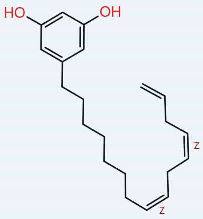	5-(8Z,11Z,14-Pentadecatrienyl)resorcinol::5-(pentadeca-8,11,14-trien-1-yl)resorcinol::5-[(8Z,11Z)-pentadeca-8,11,14-trien-1-yl]benzene-1,3-diol::CHEMBL459603::Cardol triene	60000
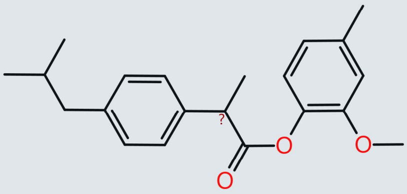	CHEMBL2262184	65000

Structure	BindingDB Ligand Name	IC50 (nM)
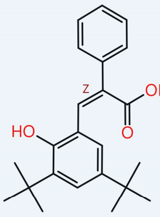	3-(3,5-di-tert-butyl-2-hydroxyphenyl)-2-phenylacrylic acid::CHEMBL228463	65000
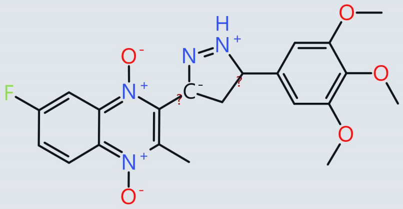	6-fluoro-2-methyl-3-[5-(3,4,5-trimethoxy-phenyl)-4,5-dihydro-1H-pyrazol-3-yl]-quinoxaline-1,4-dioxide::CHEMBL393607	70000
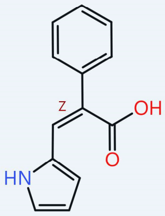	2-phenyl-3-(1H-pyrrol-2-yl)acrylic acid::CHEMBL375476	70000
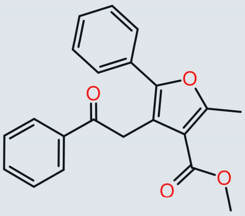	2-Methyl-4-(2-oxo-2-phenyl-ethyl)-5-phenyl-furan-3-carboxylic acid methyl ester (3a)	78500
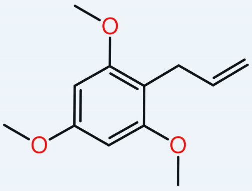	2-Allyl-1,3,5-trimethoxybenzene(7)	83200
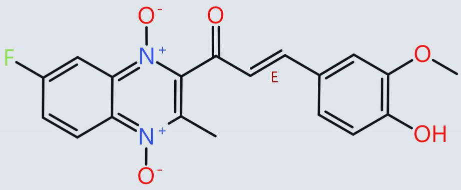	(E)-1-(7-fluoro-3-methyl-1,4-dioxy-quinoxalin-2-yl)-3-(4-hydroxy-3-methoxy-phenyl)-propenone::CHEMBL238171	85000
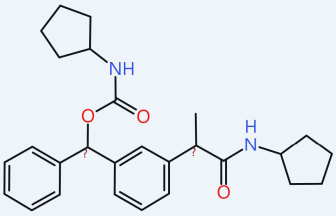	CHEMBL1172092	86000
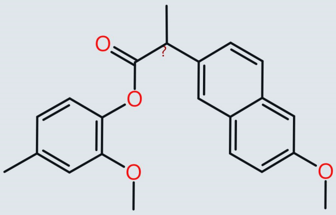	CHEMBL2262177	90000

Structure	BindingDB Ligand Name	IC50 (nM)
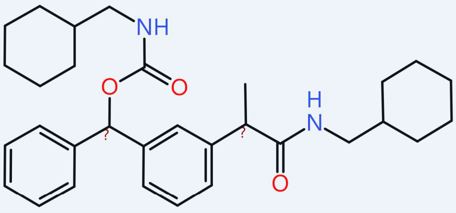	CHEMBL1170146	95000
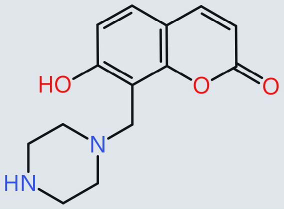	7-Hydroxy-8-piperazin-1-ylmethyl-chromen-2-one::CHEMB L364885	100000
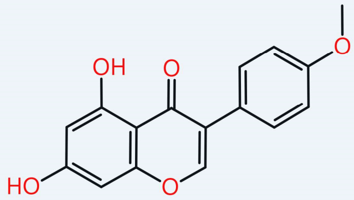	5,7-dihydroxy-3-(4-methoxyphenyl)-4H-chromen-4-one::Bio chanin A (2)::Biochanin A (BCA)::Biochanin A, 9::CHEMBL131921::cid_5280373	100000
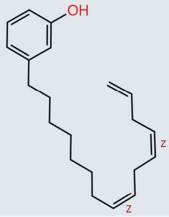	3-((8Z,11Z)-pentadeca-8,11,14-trienyl)phenol::5-{8(Z),11(Z ,14-pentadecatrienyl}phenol::CHEMBL470680::cardanol	100000
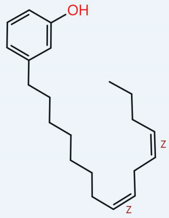	3-((8Z,11Z)-pentadeca-8,11-dienyl)phenol::5-{8(Z),11(Z)-pe ntadecadienyl}phenol::CHEMBL470887	125000
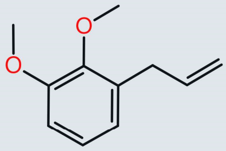	1,2-dimethoxy-3-(prop-2-en-1-yl)benzene (3)::Methylisoeugenol	128500
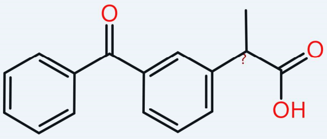	2-(3-Benzoylphenyl)propionic acid::2-(3-benzoylphenyl)propanoic acid::3-Benzoyl-alpha-methylbenzeneacetic acid::3-Benzoylhydratropic acid::CHEMBL571::Dexketoprofen trometamol::KETOPROFEN::L'Acide (benzoyl-3-phenyl)-2-propionique::Orudis (TN)::m-Benzoylhydratropic acid	130000
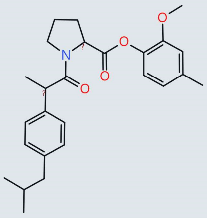	2-methoxy-4-methylphenyl 1-(2-(4-isobutylphenyl)propanoyl)pyrrolidine-2-carboxylate:: CHEMBL387544	140000

Structure	BindingDB Ligand Name	IC50 (nM)
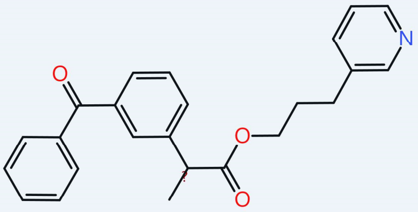	CHEMBL2262176	145000
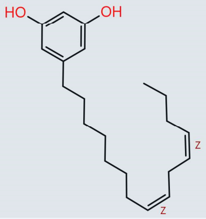	5-((8Z,11Z)-pentadeca-8,11-dienyl)benzene-1,3-diol::5-(pentadeca-8,11-dienyl)benzene-1,3-diol::5-{8(Z),11(Z)-pentadecadienyl}resorcinol::CHEMBL459604	167000
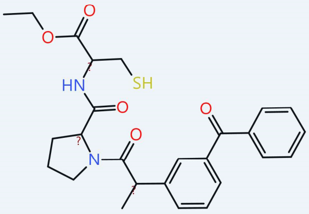	CHEMBL2262175	170000
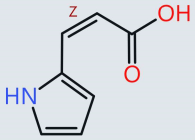	3-(1H-pyrrol-2-yl)acrylicacid::CHEMBL228464	170000
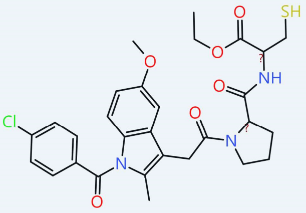	CHEMBL204505	200000
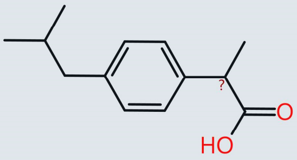	(+-)-2-(p-isobutylphenyl)propionic acid::(+-)-alpha-methyl-4-(2-methylpropyl)benzeneacetic acid::(+-)-ibuprofen::(+-)-p-isobutylhydratropic acid::(4-isobutylphenyl)-alpha-methylacetic acid::(RS)-ibuprofen::2-(4-isobutylphenyl)propanoic acid::2-[4-(2-methylpropyl)phenyl]propanoic acid::4-isobutylhydratropic acid::Advil::Brufen::CHEMBL521::IBUPROFEN	200000
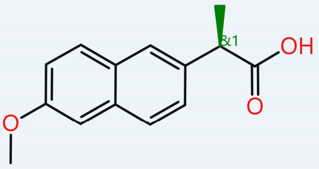	(2S)-2-(6-methoxynaphthalen-2-yl)propanoic acid::(S)-2-(6-Methoxy-naphthalen-2-yl)-propionic acid::(S)-2-(6-methoxynaphthalen-2-yl)propanoic acid::(S)-naproxen::2-(6-Methoxy-naphthalen-2-yl)-propionicacid::2-(6-Methoxy-naphthalen-2-yl)-propionic acid(naproxen)::2-(6-methoxy-2-naphthyl)propanoic acid::2-(6-methoxynaphthalen-2-yl)propanoic acid::Aleve::Anaprox::CHEMBL154::Ec-naprosyn::Equiprox	220000
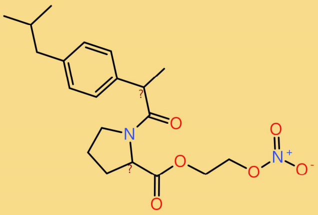	CHEMBL2262182	240000

Structure	BindingDB Ligand Name	IC50 (nM)
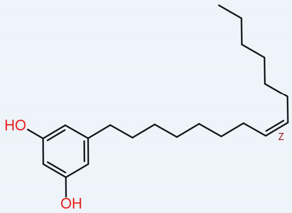	5-[(8Z)-pentadec-8-en-1-yl]benzene-1,3-diol::5-[(8Z)-pentadec-8-enyl]resorcinol::Bilobol::CHEMBL461628	250000
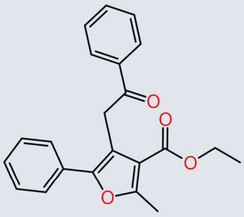	2-Methyl-4-(2-oxo-2-phenyl-ethyl)-5-phenyl-furan-3-carboxylic acid ethyl ester (3b)	287000
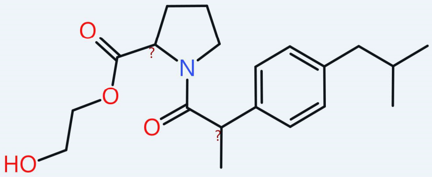	CHEMBL2262183	320000
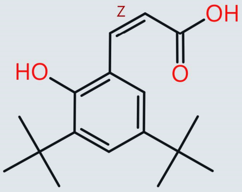	3-(3,5-di-tert-butyl-2-hydroxyphenyl)acrylic acid::CHEMBL375477	425000
